# Development of a core outcome set for therapeutic studies in eosinophilic esophagitis (COREOS)

**DOI:** 10.1016/j.jaci.2021.07.001

**Published:** 2021-07-06

**Authors:** Christopher Ma, Alain M. Schoepfer, Evan S. Dellon, Albert J. Bredenoord, Mirna Chehade, Margaret H. Collins, Brian G. Feagan, Glenn T. Furuta, Sandeep K. Gupta, Ikuo Hirano, Vipul Jairath, David A. Katzka, Rish K. Pai, Marc E. Rothenberg, Alex Straumann, Seema S. Aceves, Jeffrey A. Alexander, Nicoleta C. Arva, Dan Atkins, Luc Biedermann, Carine Blanchard, Antonella Cianferoni, Constanza Ciriza de los Rios, Frederic Clayton, Carla M. Davis, Nicola de Bortoli, Jorge A. Dias, Gary W. Falk, Robert M. Genta, Gisoo Ghaffari, Nirmala Gonsalves, Thomas Greuter, Russell Hopp, Karen S. Hsu Blatman, Elizabeth T. Jensen, Doug Johnston, Amir F. Kagalwalla, Helen M. Larsson, John Leung, Hubert Louis, Joanne C. Masterson, Calies Menard-Katcher, Paul A. Menard-Katcher, Fouad J. Moawad, Amanda B. Muir, Vincent A. Mukkada, Roberto Penagini, Robert D. Pesek, Kathryn Peterson, Philip E. Putnam, Alberto Ravelli, Edoardo V. Savarino, Christoph Schlag, Philipp Schreiner, Dagmar Simon, Thomas C. Smyrk, Jonathan M. Spergel, Tiffany H. Taft, Ingrid Terreehorst, Tim Vanuytsel, Carina Venter, Mario C. Vieira, Michael Vieth, Berber Vlieg-Boerstra, Ulrike von Arnim, Marjorie M. Walker, Joshua B. Wechsler, Philip Woodland, John T. Woosley, Guang-Yu Yang, Noam Zevit, Ekaterina Safroneeva

**Affiliations:** a Division of Gastroenterology and Hepatology, Departments of Medicine and Community Health Sciences, University of Calgary, Calgary; b Alimentiv Inc, London (Canada); c Division of Gastroenterology and Hepatology, Centre Hospitalier Universitaire Vaudois (CHUV) and University of Lausanne, Lausanne; d Center for Esophageal Diseases and Swallowing, Division of Gastroenterology and Hepatology, University of North Carolina at Chapel Hill School of Medicine, Chapel Hill; e Department of Gastroenterology and Hepatology, Amsterdam University Medical Center, Amsterdam; f Mount Sinai Center for Eosinophilic Disorders, Icahn School of Medicine at Mount Sinai, New York; g Division of Pathology and Laboratory Medicine, Cincinnati Children’s Hospital Medical Center, Cincinnati; h Department of Medicine, Western University, London (Canada); i Department of Epidemiology and Biostatistics, Western University, London (Canada); j Digestive Health Institute, Children’s Hospital Colorado, Gastrointestinal Eosinophilic Diseases Program, Section of Pediatric Gastroenterology, Hepatology, and Nutrition, University of Colorado School of Medicine, Aurora; k Division of Pediatric Gastroenterology, Hepatology, and Nutrition, Riley Hospital for Children/Indiana University School of Medicine, Indianapolis; l Division of Gastroenterology & Hepatology, Northwestern University, Feinberg School of Medicine, Chicago; m Division of Gastroenterology, Mayo Clinic, Rochester; n Department of Pathology and Laboratory Medicine, Mayo Clinic Arizona, Scottsdale; o Division of Allergy and Immunology, Department of Pediatrics, Cincinnati Children’s Hospital Medical Center, University of Cincinnati College of Medicine, Cincinnati; p Department of Gastroenterology and Hepatology, University Hospital Zurich, Zurich; q Division of Allergy Immunology, University of California, San Diego, Rady Children’s Hospital, San Diego; r Department of Pathology and Laboratory Medicine, Ann & Robert H. Lurie Children’s Hospital of Chicago, Northwestern University, Feinberg School of Medicine, Chicago; s Gastrointestinal Eosinophilic Diseases Program, Children’s Hospital of Colorado, Section of Allergy and Immunology, University of Colorado School of Medicine, Aurora; t Nestlé Institute of Health Sciences, Nestlé Research, Société des Produits Nestlé, Vevey; u Division of Allergy and Immunology, Department of Pediatrics, Children’s Hospital of Philadelphia, Perelman School of Medicine at University of Pennsylvania, Philadelphia; v Department of Gastroenterology, Hospital Clínico San Carlos, Universidad Complutense, Instituto de Investigación Sanitaria San Carlos (IdISSC), Madrid; w Department of Pathology, The University of Utah, Huntsman Cancer Hospital, Salt Lake City; x Immunology, Allergy, and Retrovirology Section of the Department of Pediatrics, Baylor College of Medicine, Texas Children’s Hospital, Houston; y Department of Translational Research and New Technology in Medicine and Surgery, Division of Gastroenterology, University of Pisa, Cisanello Hospital, Pisa; z Pediatric Gastroenterology, Centro Hospitalar S. João, Porto; aa Division of Gastroenterology, University of Pennsylvania Perelman School of Medicine, Philadelphia; bb Inform Diagnostics, Irving; cc Department of Pathology, Baylor College of Medicine, Houston; dd Division of Pulmonary, Allergy, and Critical Care Medicine, Department of Medicine, Pennsylvania State College of Medicine, Hershey; ee University of Nebraska Medical Center, Children’s Hospital and Medical Center, Omaha; ff Section of Allergy and Clinical Immunology, Dartmouth-Hitchcock Medical Center, Dartmouth Geisel School of Medicine, Hanover; gg Wake Forest University School of Medicine, Department of Epidemiology and Prevention, Winston-Salem; hh Asthma and Allergy Specialists, Charlotte; ii Division of Gastroenterology, Hepatology, and Nutrition, Department of Pediatrics, Ann & Robert H. Lurie Children’s Hospital of Chicago, Chicago; jj Division of Gastroenterology, Department of Pediatrics, John H. Stroger Jr Hospital of Cook County, Chicago; kk Department of ENT, Head, and Neck Surgery, NÄL Medical Centre, Trollhättan; ll Division of Gastroenterology, Tufts Medical Center, Boston; mm Department of Gastroenterology, Hepatopancreatology, and Digestive Oncology, Erasme Hospital, Université Libre de Bruxelles, Brussels; nn Department of Biology, Maynooth University, Kildare; oo Division of Gastroenterology and Hepatology, University of Colorado Anschutz Medical Campus, Aurora; pp Division of Gastroenterology & Hepatology, Scripps Clinic, La Jolla; qq Center for Pediatric Eosinophilic Diseases, Division of Gastroenterology and Hepatology & Nutrition, Children’s Hospital of Philadelphia, University of Pennsylvania Perelman School of Medicine, Philadelphia; rr Division of Gastroenterology, Hepatology, and Nutrition, Cincinnati Children’s Hospital Medical Center, Department of Pediatrics, University of Cincinnati College of Medicine, Cincinnati; ss Department of Pathophysiology and Transplantation, University of Milan, Milan; tt Fondazione IRCCS Ca’ Granda, Ospedale Maggiore Policlinico, Milan; uu Division of Allergy and Immunology, University of Arkansas for Medical Sciences and Arkansas Children’s Hospital, Little Rock; vv Division of Gastroenterology, The University of Utah, Salt Lake City; ww University Department of Pediatrics, Children’s Hospital–Spedali Civili, Brescia; xx Department of Surgery, Oncology, and Gastroenterology, DiSCOG, University of Padua, Padua; yy II. Medizinische Klinik, Klinikum Rechts der Isar, Technische Universität München, Munich; zz Department of Dermatology, Inselspital, Bern University Hospital, University of Bern, Bern; aaa Department of Pathology, Mayo Clinic, Rochester; bbb Department of ENT, Amsterdam University Medical Centre, Amsterdam; ccc Gastroenterology and Hepatology, University Hospitals Leuven, Leuven; ddd Translational Research in Gastrointestinal Disorders, KU Leuven, Leuven; eee Department of Pediatrics, Pontifical Catholic University of Paraná and Center for Pediatric Gastroenterology, Hospital Pequeno Príncipe, Curitiba; fff Institute for Pathology, Klinikum Bayreuth, Friedrich-Alexander-University Erlangen-Nuremberg, Erlangen; ggg OLVG Hospital, Department of Pediatrics, Amsterdam; hhh Department of Gastroenterology, Hepatology, and Infectious Diseases, University Hospital, Magdeburg; iii Centre of Research Excellence in Digestive Health, University of Newcastle, Newcastle; jjj Wingate Institute of Neurogastroenterology, Barts, and the London School of Medicine and Dentistry, Queen Mary University of London, London (UK); kkk Department of Pathology and Laboratory Medicine, University of North Carolina at Chapel Hill School of Medicine, Chapel Hill; lll Division of Pathology, Northwestern University, Feinberg School of Medicine, Chicago; mmm Institute of Gastroenterology, Nutrition, and Liver Diseases, Schneider Children’s Medical Center of Israel, Petach Tikva; nnn Sackler Faculty of Medicine, Tel Aviv University, Tel Aviv; ooo Institute of Social and Preventive Medicine, University of Bern, Bern.

**Keywords:** Eosinophilic esophagitis, outcomes, clinical trials, end points, histology, histopathology, endoscopy, symptoms, patient-reported outcomes, quality of life

## Abstract

**Background:**

End points used to determine treatment efficacy in eosinophilic esophagitis (EoE) have evolved over time. With multiple novel therapies in development for EoE, harmonization of outcomes measures will facilitate evidence synthesis and appraisal when comparing different treatments.

**Objective:**

We sought to develop a core outcome set (COS) for controlled and observational studies of pharmacologic and diet interventions in adult and pediatric patients with EoE.

**Methods:**

Candidate outcomes were generated from systematic literature reviews and patient engagement interviews and surveys. Consensus was established using an iterative Delphi process, with items voted on using a 9-point Likert scale and with feedback from other participants to allow score refinement. Consensus meetings were held to ratify the outcome domains of importance and the core outcome measures. Stakeholders were recruited internationally and included adult and pediatric gastroenterologists, allergists, dieticians, pathologists, psychologists, researchers, and methodologists.

**Results:**

The COS consists of 4 outcome domains for controlled and observational studies: histopathology, endoscopy, patient-reported symptoms, and EoE-specific quality of life. A total of 69 stakeholders (response rate 95.8%) prioritized 42 outcomes in a 2-round Delphi process, and the final ratification meeting generated consensus on 33 outcome measures. These included measurement of the peak eosinophil count, Eosinophilic Esophagitis Histology Scoring System, Eosinophilic Esophagitis Endoscopic Reference Score, and patient-reported measures of dysphagia and quality of life.

**Conclusions:**

This interdisciplinary collaboration involving global stakeholders has produced a COS that can be applied to adult and pediatric studies of pharmacologic and diet therapies for EoE and will facilitate meaningful treatment comparisons and improve the quality of data synthesis.

Eosinophilic esophagitis (EoE) is a chronic immune-mediated disease characterized histologically by esophageal eosinophil–predominant inflammation and clinically by symptoms of esophageal dysfunction.^1^ Since its initial description in the early 1990s, there has been a significant increase in the incidence of EoE; prevalence rates from population-based studies estimate that approximately 50 to 100 per 100,000 persons are affected.^2^ The diagnosis of EoE is based on both symptoms consistent with esophageal dysfunction, particularly dysphagia in adolescents and adults, and the presence of histologic inflammation, defined as a peak eosinophil count (PEC) of ≥15 eosinophils per high-power field (eos/hpf), with exclusion of other causes of esophageal eosinophilia.^3^ Untreated EoE can progress to the development of fibrostenotic complications such as strictures and endoscopically impassable rings, which are associated with progressive symptoms, food impaction, and poor quality of life (QoL).^4–6^

Consensus treatment recommendations for EoE have historically included: (1) elimination diets that restrict exposure to potential food allergens, (2) endoscopic dilation for fibrostenotic complications, (3) proton pump inhibitors, and (4) swallowed topical corticosteroids that reduce eosinophilic inflammation.^7,8^ However, these approaches have inherent limitations. Patients must adhere to substantial lifestyle changes for dietary strategies to be effective, dilation carries procedural risks and does not address the underlying inflammatory pathophysiology, and proton pump inhibitors are not effective in all EoE patients. A lack of approved esophageal-specific formulations in many jurisdictions, potential treatment-related adverse effects, and short duration of efficacy limit the potential of using swallowed topical corticosteroids in the long term for managing a chronic disease that almost universally recurs after treatment cessation.^9–11^ Accordingly, there has been tremendous interest in developing EoE-specific pharmacotherapies,^12^ with over 50 active or enrolling interventional studies for the treatment of EoE registered at ClinicalTrials.gov. Furthermore, recent positive results from phase 3 trials of dupilumab, a monoclonal antibody targeting the IL-4 receptor alpha, budesonide orodispersible tablets as both induction and maintenance therapy, and budesonide oral suspension have inspired even greater enthusiasm for drug development in this field.^13–16^

Despite these breakthroughs, a major limitation to efficient drug development in EoE has been the lack of standardized outcome measures for use in both registrational trials that can support labeling claims and in observational studies that can answer practice-based questions.^17^ Although validated, reliable, and responsive instruments of EoE disease activity exist,^18–27^ agreement on the most appropriate end points for use in clinical studies has not been reached, and significant heterogeneity exists in the outcome measures that are reported.^28^ Given the lack of consensus and the increasing scrutiny on outcome measures in clinical trials of EoE, developing a core outcome set (COS) is a research priority. A COS is a consensus-derived minimum set of outcomes that should be measured and reported in all trials in a given therapeutic area.^29^ COS development focuses on identifying relevant and appropriate end points through an iterative, data-driven process involving all major stakeholders, including researchers, clinicians, and patients. Advantages of adopting a COS include improving the efficiency of clinical studies by ensuring appropriate end points are measured, minimizing heterogeneity in outcome reporting, reducing risk of publication bias, improving the quality of evidence synthesis, and facilitating fair comparisons across different therapies.

Therefore, in collaboration with the Consortium of Eosinophilic Gastrointestinal Disease Researchers (CEGIR) and the European Society of Eosinophilic Esophagitis (EUREOS), as well as individuals recruited from the Eosinophil Gastrointestinal Disorders (EGID) committee of the American Academy of Allergy, Asthma & Immunology (AAAAI), we aimed to develop an international consensus COS for use in studies of pharmacologic and dietary interventions for adult and pediatric patients with EoE (COREOS).

## METHODS

### Scope and protocol registration

The COREOS initiative is registered with Core Outcome Measures in Effectiveness Trials (COMET) (www.comet-initiative.org) and was conducted in accordance with the guidelines outlined in the COMET handbook and the standards established by the Core Outcome Set–Standards for Development.^29,30^ This article was drafted on the basis of the Core Outcome Set–Standards for Reporting statement.^31^ The patient study was approved by the ethics committee at the University of Lausanne (CER-VD 148/15).

The scope of this COS is to include all pharmacologic and dietary therapies, in both controlled trials and observational studies, for pediatric and adult patients with EoE. Although endoscopic dilation is an important component of management for patients with EoE, the measurement of treatment success after dilation, including procedural and technical success, is fundamentally different from evaluating therapeutic efficacy of pharmacologic or dietary strategies. We evaluated outcomes for observational studies separately from those in controlled trials, which are typically conducted in different settings, using different methods, and with different levels of study funding and logistical support. These factors are relevant for the feasibility of measuring certain outcomes.

### Overview of COS development

**FIG 1.**
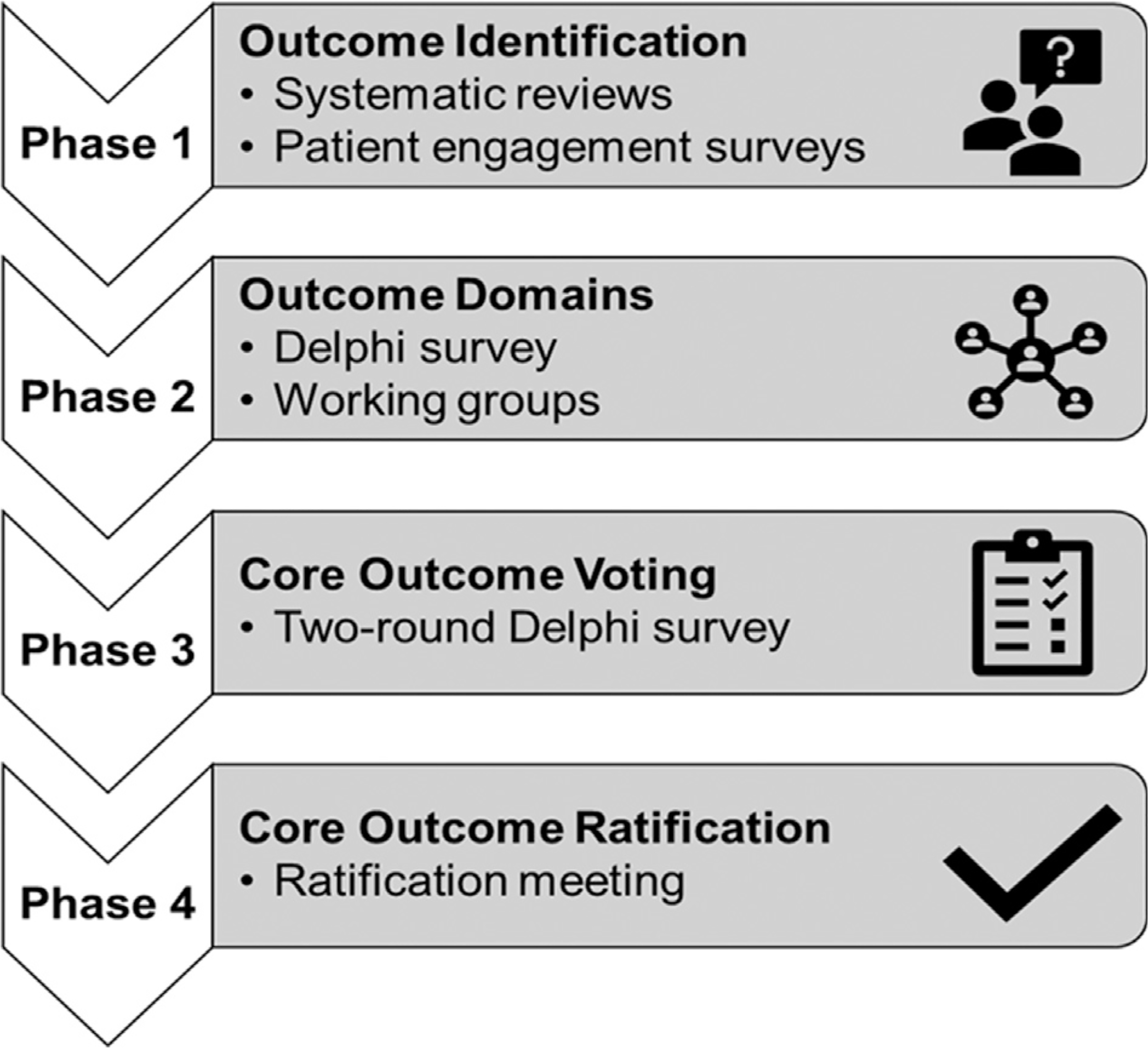
Core outcome set development process.

The COS was developed using a multiphase approach summarized in Fig 1. First, systematic reviews of the literature and patient engagement surveys were conducted to identify candidate outcomes that have been previously measured and/or are important to patients with EoE. Next, we used this information to build a framework of different outcome domains. Working groups for each domain were assembled to review the literature for relevant end points, and a Delphi survey was conducted to categorize these domains into core, important, and research agenda domains that were based on the Outcome Measures in Rheumatology model.^32^ Core outcome domains were carried forward into the next phase. In phase 3, a comprehensive list of outcome measures within each of the core domains was evaluated by a panel of multidisciplinary experts in a 2-round Delphi survey to establish consensus. Finally, a virtual ratification meeting was held to vote on the final outcomes included in the COS.

### Participants

We gathered input from a diverse range of adult and pediatric patients with EoE to determine their values and opinions on the importance of different outcomes.^33^ Patients and caregivers of pediatric patients were recruited using purposive sampling from multiple clinics to capture a range of disease duration, disease activity (including both symptomatic and asymptomatic patients), disease experiences, and treatment experiences (including patients who had previously been exposed to proton pump inhibitors, swallowed topical corticosteroids, dilation, and dietary exclusion). We focused on engaging patients early in phase 1 of this COS development to determine the appropriate outcome domains for measurement.

In phases 2 and 3, we targeted a minimum sample size of 50 respondents for each Delphi survey. A diverse participant pool was identified and invited by the lead and senior investigator, and included gastroenterologists, pathologists, allergists, researchers, dieticians, psychologists, and methodologists. Selected participants reflected a broad range of clinical knowledge and geographical experience. Panelists were required to have expertise in EoE, demonstrated by peer-reviewed publications or clinical experience in managing adult or pediatric EoE patients.

### Phase 1: Outcome identification

Three systematic reviews were conducted to ensure that we comprehensively evaluated the literature with respect to the scope of this COS: (1) a systematic review to assess the operating properties of evaluative indexes used in EoE;^34^ (2) a systematic review to assess the outcome measures used in randomized controlled trials (RCTs) in EoE;^28^ and (3) a systematic review to assess the outcome measures used in observational studies in EoE (including studies of topical corticosteroids, dietary measures, and endoscopic dilation).^35^ In addition, a systematic review to assess the outcome measures used in pediatric RCTs was previously published by Rubin et al.^36^ Although dilation was outside the scope of this COS, we specifically searched for outcomes used in studies of endoscopic dilation to ensure that potentially relevant end points were not missed. In summary, searches were conducted in Medline, Embase, the Cochrane Central Register of Controlled Trials (CENTRAL), ClinicalTrials.gov, and the EU Clinical Trials Register to identify relevant studies. Evaluative indexes and outcomes used to measure treatment efficacy were identified.

Swiss patients with EoE were engaged to identify their perspective on relevant outcomes for measurement.^33^ Patient participation consisted of semistructured interviews and paper-based surveys aimed at assessing the relative importance of different treatment goals and outcome measures in EoE. Semistructured interviews were conducted with EoE patients and used to create a patient survey list of short- and long-term outcomes of importance for therapeutic efficacy. The survey was then distributed to patients with EoE to determine the ranked importance of different outcomes in the following domains: symptoms, QoL, endoscopy, and histology.

### Phase 2: Outcome domains

The information identified from the systematic reviews and patient engagement surveys was used to construct a framework of 11 outcome domains. A Delphi survey was distributed to all experts to identify which domains were of importance to include in the COS. Each domain was ranked on a 9-point Likert scale, based on the Grading of Recommendations Assessment, Development, and Evaluation working group definitions.^37^ Scores of 1 to 3 indicate an outcome domain that was not considered important for inclusion, scores of 4 to 6 indicate an outcome domain that was considered important but not critical for inclusion, and scores of 7 to 9 indicate an outcome domain thought to be critical for inclusion in the COS. An option to select “unsure of significance or unable to score” was also available. *A priori,* outcome domains scored in the 7–9 range by ≥70% of panelists and in the 1–3 range by <15% of panelists were carried forward to phase 3 as core domains. Working groups consisting of experts in each domain were organized and met by teleconference to review the relevant end points. These outcome domains were discussed at a moderated in-person meeting that occurred at Digestive Disease Week 2019 (San Diego, Calif). Outcomes that did not meet the threshold for core domains were reviewed, and those with limited available evidence on their use in EoE were assigned as research agenda domains.

### Phase 3: COS voting

A comprehensive list of outcomes identified within each core domain, as well as measurement tools and definitions, were included in an online 2-round Delphi survey. Participants were asked to rank each outcome on a 9-point Likert scale as described above, with a specific focus on ranking the most important outcomes for inclusion. Free-text entry was available so participants could provide clarification, suggest wording changes, recommend additional end points, or provide compelling rationale and arguments for inclusion or exclusion of certain items. Each round was open for 8 weeks to ensure all participants had adequate time to complete the survey.

Responses from the first round were analyzed and collated into a feedback report. Descriptive statistics were used to summarize the number of participants scoring each outcome and the distribution of scores. All open-ended responses were reviewed by the lead and senior investigators to evaluate substantial arguments and additional suggestions. Responses from round 1 were used to determine the outcomes carried forward to round 2 based on rules established *a priori.* Outcomes scored in the 7–9 range by ≥50% of the panelists and 1–3 range by <15% of the panelists were carried forward. These definitions have been previously used in COS exercises and were aimed at mitigating the risk of panelist fatigue.^29^ All panelists who completed the round 1 survey were invited to participate in round 2 and received an individualized feedback report summarizing both their initial voting results and the results from the group. Panelists were then asked to rescore each outcome on the same 9-point Likert scale, with consideration based on insights from the group. Outcomes scored in the 7–9 range by ≥70% of the panelists and in the 1–3 range by <15% of the panelists were decided to have met consensus for inclusion. Outcomes scored in the 1–3 range by ≥70% of the panelists and in the 7–9 range by <15% of the panelists were defined to have met consensus for exclusion.

We recognize that it is implausible for any single panelist to be completely familiar with every scoring system/grading tool evaluated in this consensus. This was mitigated by choosing a multidisciplinary panel, instructing panelists to not answer questions with which they were unfamiliar, and basing consensus definitions on the proportion of respondents. Analysis of missing data suggests that specialists performing endoscopy drove decisions for endoscopic findings, specialists following adult patients drove decisions for symptoms and QoL outcomes in adults, and specialists following pediatric patients drove decisions for symptoms and QoL outcomes in pediatric populations.

### Phase 4: Final COS ratification and consensus definitions

A moderated teleconference to ratify the final COS was conducted December 8, 2020. Although this was initially planned as a face-to-face meeting with all stakeholder groups to discuss all items from the round 2 survey, this was amended to a virtual meeting as a result of coronavirus disease 2019 public health restrictions. We elected to discuss only those items that had a reasonable likelihood of being included in the COS: assuming a binomial distribution, outcomes for which the upper 95% confidence interval of the proportion of panelists voting in the 7–9 category exceeded 70% were carried forward to discussion in the ratification meeting. Logistically, it was infeasible for every panelist voting in the Delphi surveys to participate in the ratification teleconference, given the international participation; however, as per the COMET recommendations, representatives from every discipline were present, and the ratification panel was similar in composition to the Delphi panelists. Panelists were shown the results from round 2 voting, and the criteria for inclusion were reviewed. All items, including those with consensus, were discussed to ensure that any compelling arguments for or against inclusion were heard and reviewed. After discussion, panelists voted on items anonymously. In this ratification round, voting was simplified to “include in the COS,” “do not include in the COS,” or “unsure.” Items receiving ≥70% of votes in the “include in the COS” category and <15% of votes in the “do not include in the COS” category were ratified for final inclusion.

## RESULTS

### Participants

A total of 36 adult patients with EoE participated in the semistructured interviews, and paper-based surveys were completed by 109 (73.6%) of 148 patients.^33^ The mean ± SD age was 50.2 14.5 years, with a disease duration of 7.7 ± 4.7 years. Seventy-eight percent of patients (85/109) were male, and approximately one third (33.9%, 37/109) had previously experienced a food bolus impaction. A total of 30.3% (33/109) of patients were receiving proton pump inhibitors, 62.4% (68/109) were receiving swallowed topical corticosteroids, and 11.0% (12/109) were receiving elimination diets. Pediatric patients and their caregivers were separately surveyed: 30 patients aged >11 years and 15 patients aged <11 years were included. Among pediatric patients, 80.0% (36/45) had associated atopic conditions, 71.4% (25/35) were treated with swallowed topical corticosteroids, and 25.7% (9/35) were receiving an elimination diet.

Demographic characteristics of the expert panelists in each of the Delphi rounds are summarized in Table I. Members of CEGIR and EUREOS, and individuals recruited from the EGID committee of AAAAI were invited to participate in COREOS exercise. A total of 66, 69, and 62 experts participated in the outcome domains survey, round 1 COS survey, and round 2 COS survey, respectively. The response rates were 95.8% (69/72) and 89.9% (62/69) for round 1 and 2 surveys, respectively. Twenty-seven participants attended the phase 4 ratification videoconference. Across all rounds, there were participants from multiple specialties and 16 different countries.

### Phase 1: Outcome identification systematic reviews and patient engagement

Detailed results from the systematic reviews have been previously published; the major findings are summarized here. In the first review of disease activity indexes and their operating properties, 4373 citations were evaluated to identify 130 eligible studies. The Adult Eosinophilic Oesophagitis Quality of Life questionnaire, Eosinophilic Esophagitis Histology Scoring System (EoEHSS), Eosinophilic Esophagitis Endoscopic Reference Score (EREFS), symptom-based Eosinophilic Esophagitis Activity Index (EEsAI) Patient-Reported Outcome (PRO) instrument, Dysphagia Symptoms Questionnaire (DSQ), Pediatric Eosinophilic Esophagitis Symptom Score (PEESS v2.0), and Pediatric Quality of Life Inventory (PedsQL) EoE were identified as indexes that were either reliable, responsive, or valid measures of disease activity.^34^ In a second review of outcome measures used in RCTs, 22 placebo-controlled trials including 1112 patients with EoE were evaluated, with substantial heterogeneity in the definitions of histologic, endoscopic, and PRO-based response and remission.^28^ The use of histologic end points was associated with the lowest rate of placebo response.

A third review of outcome measures used in observational studies (including cohort, case series, randomized open-label trials, and case–control studies) of adults with EoE was conducted.^35^ A total of 69 studies were included. Histologic, endoscopic, and patient-reported symptom-based end points were the most frequently reported, although no consistent definitions of response or remission were identified. Esophageal eosinophil density was the most frequently reported outcome (in 60 studies), with varying thresholds for response/remission ranging from 5 to 15 eos/hpf. Endoscopic outcomes were assessed in 44 studies, although a formal scoring system such as the EREFS was not routinely used. Similarly, there was substantial heterogeneity in instruments used for measuring symptom-based responses. In addition to the EEsAI and DSQ, other tools that have been used included the Mayo Dysphagia Questionnaire, Dysphagia Frequency Scale, Watson Dysphagia Score, Straumann Dysphagia Index, and multiple nonvalidated *ad hoc* scores based on different combinations of the frequency, intensity, and/or duration of dysphagia, food bolus impaction, abdominal or chest/retrosternal pain, heartburn, regurgitation, and/or lifestyle modifications.

In the patient engagement surveys, patients considered improvement in EoE-related symptoms and QoL as the most important end points: over 90% of patients chose improvement in symptoms and disease-specific QoL as highly important outcomes both in the short and long term. Reductions in endoscopic and histologic inflammation were also considered important outcomes, although more so in the long term rather than the short term (89.9% vs 72.9% for endoscopic and 81.3% vs 61.7% for histologic outcomes, respectively).^33^ Among pediatric patients, over 90% of both caregivers and patients ranked symptom and QoL improvement as important short- and long-term therapeutic goals, and over 80% attributed importance to achieving short- and long-term histologic end points.

### Phase 2: Outcome domains

**FIG 2.**
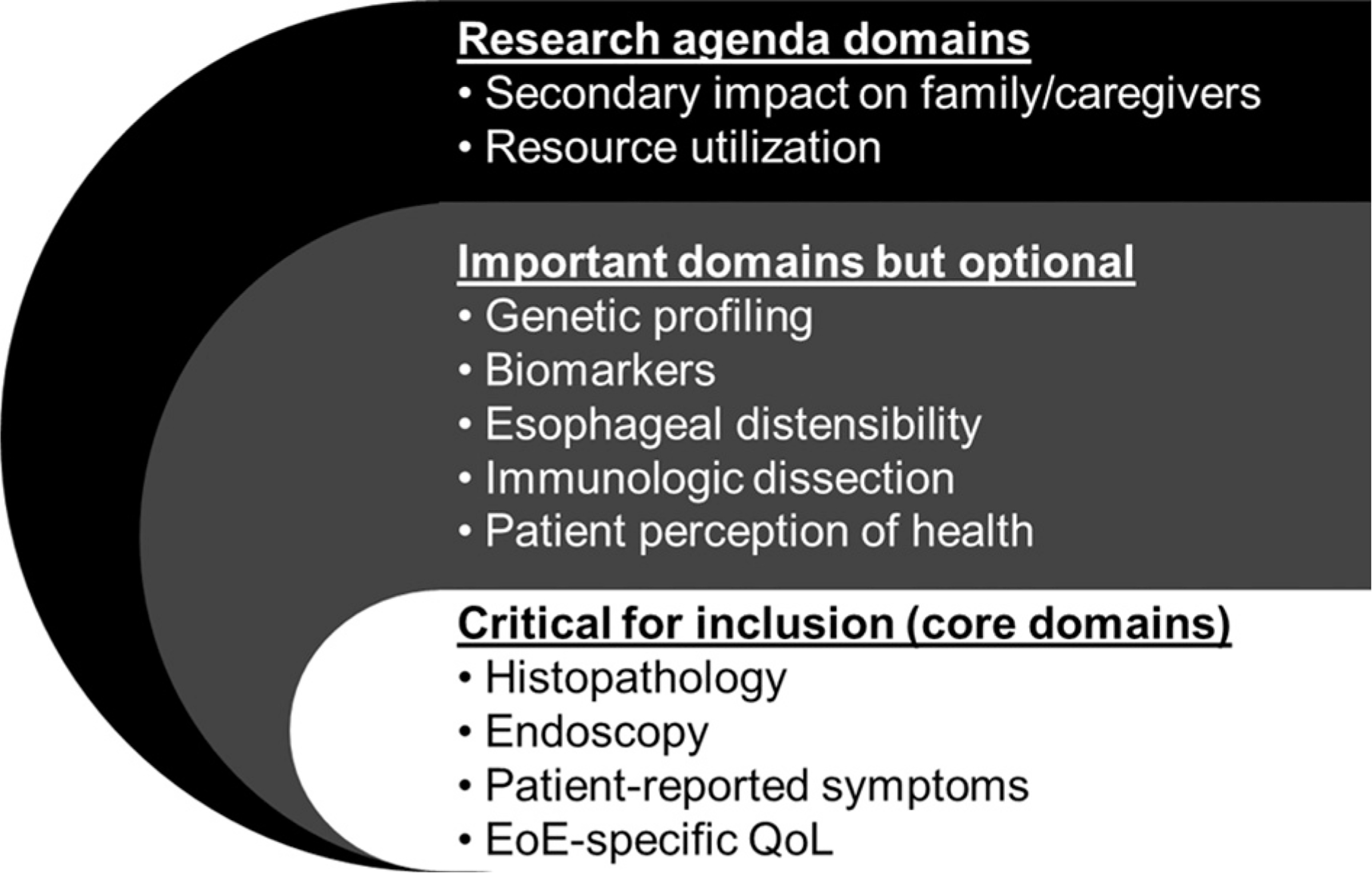
Outcome domains for inclusion in the eosinophilic esophagitis core outcome set.

Using the information from phase 1, we created a framework of 3 major categories of outcome domains: (1) clinician-reported domains (including histopathology, endoscopy, esophageal distensibility, immunologic dissection, genetic profiling, and biomarkers); (2) patient-reported domains (including patient-reported symptoms, patient-reported QoL, and patient perception of health); and (3) other domains (including secondary impact on caregivers and resource utilization). The importance of each domain for inclusion in a COS was reviewed in working groups and then in a face-to-face meeting. A Delphi survey was then distributed to expert panelists, and 4 outcome domains were voted as critical for inclusion (Table II and Fig 2): patient-reported symptoms, EoE-specific QoL, histopathology, and endoscopy. The other domains were considered either important but optional at this time, or domains for the research agenda that require additional investigation.

### Phase 3: COS voting

A total of 122 items across the 4 core outcome domains were included in the round 1 Delphi survey, which was completed by 69 panelists. Results from round 1 survey are summarized in Table E1 in this article’s Online Repository at www.jacionline.org. These items were organized by outcome domain (58 items for histopathology, 28 items for endoscopy, 24 items for patient-reported symptoms, and 12 items for EoE-specific QoL) and stratified by study type (RCTs vs observational studies) and patient population (adult vs pediatric). All free-text responses were reviewed and incorporated into the second round of voting. A total of 59 outcomes (18 for histology, 12 for endoscopy, 19 for patient-reported symptoms, and 10 for EoE-specific QoL) were included in the round 2 survey. Results from round 2 survey are summarized in Table E2 in this article’s Online Repository at www.jacionline.org.

### Phase 4: Ratification meeting and COS

A total of 42 items from the round 2 survey were discussed and voted on in the ratification meeting, and 2 additional items were introduced after panel discussion. After voting, 33 items were included in the final COS (Table III).

#### COS: Histopathology outcomes.

With respect to histopathology outcomes, there was consensus that the PEC should be reported in all RCTs and observational studies, expressed either as eos/hpf (including exact area used and the hpf size reported in square millimeters) or as eosinophils per square millimeter (eos/mm^2^), viewed at 400× magnification. Several panelists identified that both measures should be reported, as eos/hpf has historically been used in the literature, whereas eos/mm^2^ adjusts for potential differences in microscope ocular field size. There was consensus that histologic remission should be reported in all studies. However, the precise threshold for histologic remission was debated. There was consensus that the proportion of patients with <15 eos/hpf in all esophageal locations should be reported in both RCTs and observational studies; there was no consensus on using a more stringent threshold of ≤6 eos/hpf, even for RCTs. In RCTs, the EoEHSS should be used, and both the grade and stage of each component item should be reported.

#### COS: Endoscopy outcomes.

The panel voted that the EREFS should be used in both RCTs and observational studies to standardize endoscopic assessment of EoE disease activity, scoring the most severe grade of EoE-associated features. Additionally, both inflammatory and fibrotic components of the EREFS should be reported. In the round 1 survey, different versions of the EREFS were explored: (1) scoring from 0 to 9 as originally proposed; (2) scoring from 0 to 8 (with furrows scored as absent/present); (3) scoring from 0 to 16 (a 0–8 score summed for 2 different esophageal locations); and (4) scoring from 0 to 18 using alternative weighting of the different components. Following the *a priori*–defined rules for moving items to the next round, only the EREFS scores from the 0–8 group were carried forward to round 2 because of a higher proportion of panelists voting to not include other versions of the EREFS. However, there was extensive discussion that scoring on a 0–8 scale may result in a narrower dynamic range of the EREFS score and decrease responsiveness measured by endoscopy. Additionally, if scoring is performed on a 0–9 scale, *post hoc* analysis collapsing the categories for moderate-to-severe furrows can generate an EREFS score on a 0–8 scale, but not vice versa. In an *ad hoc* vote, 14 (66.7%) of 21 panelists favored using the EREFS from 0–9, whereas 7 (33.3%) of 21 panelists favored using the EREFS 0–8 scale. Given that this voting was held outside the defined methods of COS development, reporting the original EREFS is optional if the individual components are provided, so that readers can collapse the furrows’ grading to generate a comparable score on the 0–8 scale. For both RCTs and observational studies, there was consensus that endoscopic remission should be defined on the basis of the EREFS using a cutoff of ≤2. It is worth keeping in mind that although the endoscopic EREFS-based remission definition as an EREFS score of ≤2 was derived on the basis of EREFS scoring from 0 to 8 and from 0 to 9, the endoscopic inflammatory EREFS-based remission defined as the inflammation-associated components (exudate, edema, furrows) score of ≤2 is based on EREFS scoring from 0 to 8.

#### COS: Patient-reported symptoms.

There was consensus that validated instruments for patient-reported symptoms, including the DSQ and the EEsAI, should be assessed in EoE RCTs. However, there was discussion that the initial rounds of the Delphi surveys were completed before guidance was released by the US Food and Drug Administration (FDA) that highlighted the use of clinical outcome assessment instruments that use daily assessments. The EEsAI was developed and has previously been used in RCTs with a 7-day recall period as a secondary end point, and this outcome was voted to be included in the COS, recognizing that there was preference from the FDA for use of an instrument with a 24-hour recall period. The 24-hour EEsAI was added as an item for voting thanks to the discussion, but it did not meet the criteria for consensus (see Table E3 in this article’s Online Repository at www.jacionline.org). There was also consensus that the language used to query dysphagia in adults with EoE include “trouble swallowing” and “delayed/slow passage of food.” While “food being stuck” did meet the consensus thresholds in round 2 of the Delphi voting, it did not reach consensus thresholds in the ratification round, as experts identified that this should be more appropriately used for defining food bolus obstruction. No instruments for measuring symptom severity reached consensus for use in observational studies.

Separate instruments were considered for pediatric patients. In pediatric trials, there was consensus that symptoms should be measured using the PEESS v2.0 for RCTs, but not for observational studies.

#### COS: Quality of life.

There was consensus that QoL should be measured in EoE RCTs using the EoE-QoL-A for adults and the PedsQL EoE module for pediatrics. When using the PedsQL EoE module, it was considered appropriate for both parent proxy report and child self-report to be reported in RCTs. The panel discussed that it was ideal to use disease-specific QoL measures rather than generic QoL measures for this domain. No instruments for use in all observational studies met the consensus threshold for inclusion in the COS.

## DISCUSSION

In this multidisciplinary, international collaboration between multiple stakeholder groups, we developed a COS to standardize outcome reporting in therapeutic studies of pharmacologic and diet interventions in EoE. We identified 4 critical outcome domains (histopathology, endoscopy, patient-reported symptoms, and EoE-specific QoL) that are important to patients, clinicians, and researchers and that reflect the clinicopathologic hallmarks of the disease. Through multiple group discussions and several rounds of voting, we identified measurement tools that should be used to standardize disease activity assessment, both in controlled and observational studies. We took into consideration the appropriateness and validity of different end points, feasibility of measurement, and relative importance of different outcomes to each stakeholder. The application of this COS should improve the quality of research in EoE and serve as an impetus for improving clinical care by encouraging clinicians to assess core outcomes of treatment success.

This COS will be directly applicable to RCTs of novel therapies currently in development for EoE. However, the panel recognized that important elements of trial design, including outcome selection, will depend on who is conducting the trial (investigator vs industry initiated) and the subsequent regulatory requirements for labeling claims. During the development of this COS, the FDA released guidance for EoE clinical trials.^38^ Key takeaways included the selection of EoE-related symptoms and histology as co–primary end points, use of a clinical outcome assessment instrument based on daily recall, and defining histologic remission based on having ≤6 eos/hpf in all biopsy samples. The similarities, but also differences, between FDA guidance and our independent recommendations are notable. Although the COS does not precisely map onto this regulatory guidance, our framework of measuring patient-reported symptoms and histopathology as core domains is complementary, and also extends to observational studies. Moreover, we included EoE-specific QoL as an important domain of measurement, particularly for patients, and endoscopic assessment as not only an important tool for clinicians to directly visualize the esophageal mucosa but also a pre-requisite to obtaining biopsy samples.

Given the importance of eosinophilic inflammation in defining EoE, it was not surprising that histopathology was almost universally agreed on as a core domain. However, 3 areas of controversy garnered more discussion. First, the panel reviewed the reporting of peak eosinophil density based on eos/hpf versus eos/mm^2^. Although using eos/mm^2^ was thought to be advantageous for standardizing density measurements across different microscopes and field sizes,^39^ most of the literature to date has expressed the PEC per hpf, and there was consensus that this should continue to be measured and reported to facilitate historical treatment comparisons and ensure interpretability. However, the panel thought it was feasible to report both measures and recognized that particularly for RCTs, standardization of field size analysis was crucial to achieve. Therefore, we advocate for a greater emphasis on reporting eos/mm^2^ (using remission definitions of PEC ≤25 eos/mm^2^ and <60 eos/mm^2^, corresponding to PEC of ≤6 eos/hpf and <15 eos/hpf, respectively).

Second, there was consensus that a PEC of <15 eos/hpf should be used as the threshold to define histologic remission, although this is discordant from the FDA recommendations. Historically, multiple cutoff points have been used to define EoE, ranging from 5 to 30 eos/hpf.^40^ However, the data to support the use of these cutoffs are scarce. Reed et al^41^ compared different histologic cut points for treatment response: whereas a threshold of <15 eos/hpf was attainable in most patients and identified patients with endoscopic improvement, a lower cutoff of <5 eos/hpf best predicted combined symptomatic and endoscopic response. At present, the patients in clinical practice reaching histologic remission defined by <15 eos/hpf do not typically undergo therapeutic escalation to reach the target of ≤6 eos/hpf. However, a formal prospective blinded RCT examining the utility of different treatment targets is needed to answer the clinical question of whether remission should be targeted at either −≤6 or <15 eos/hpf, and whether maintenance of these treatment targets results in better outcomes for patients, including less strictures and impactions. Several guidelines since 2007 have now established ≥15 eos/hpf as the cutoff for diagnostic purposes, and the panel voted that the proportion of patients experiencing a PEC lower than this threshold should continue to be reported.^3,42,43^ Finally, the panel identified that a threshold of ≤6 eos/hpf may be too rigorous to achieve and may not necessarily be appropriate for potential future drug targets with mechanisms of action that do not directly inhibit eosinophils (for example, antifibrotic therapies). Nevertheless, we anticipate that in future trials designed for regulatory approval of medications, the proportion of patients with posttreatment PECs of <15 eos/hpf and ≤6 eos/hpf will both be reported.

Finally, there was discussion regarding the use of the EoEHSS as a measure of histologic disease activity. The EoEHSS has been previously demonstrated to be valid, reliable, responsive, and applicable in adult and pediatric populations; in addition, it correlates with other measures of disease activity, including patient symptoms, and measures histologic items that are prevalent in patients with EoE beyond the PEC alone.^21,22,24,44–47^ For these reasons, panelists strongly thought that the EoEHSS should be routinely evaluated in RCTs. However, panelists did not include the EoEHSS as a core outcome in observational studies as a result of concerns about the time required for interpretation, complexity of the score, and lack of an atlas to help pathologists not specialized in EoE to score some of the features.

There was consensus that endoscopic end points should be reported in all EoE studies and that the EREFS should be used to standardize endoscopic evaluation. The EREFS score has been shown to accurately identify disease activity in both adult and pediatric populations,^48^ can be reliably scored by experts and quickly learned by nonexperts,^18,49^ and is responsive to treatment.^24,50,51^ However, there was debate whether the EREFS should be scored on a 0–9 or 0–8 scale (depending on the grading of linear furrows), recognizing that scoring on a broader range may improve the sensitivity of the instrument for detecting change after treatment and can be converted to a 0–8 scale *post hoc* if required. Although two thirds of the ratification panel was in favor of reporting the EREFS using a 0–9 scale, consensus on the 0–8 scale was included in the COS for methodologic consistency. Functionally, reporting individual component subscores of the EREFS and grading furrows on a 3-point rather than binary scale nullifies this dilemma, and is also required to discern endoscopic inflammatory versus fibrostenotic disease activity.

Although both the DSQ and symptom-based EEsAI PRO (7-day recall period) instruments were recommended for use in RCTs of adults with EoE, there were concerns that US regulatory authorities have specifically recommended the use of an instrument with a 24-hour recall period. The DSQ was the only 24-hour recall instrument selected out of a myriad of options and is the first such instrument to be validated for use in RCTs, allowing assessment of end points such as dysphagia-free days.^14,23,38,51,52^ Other instruments, including both conceptually similar and dissimilar tools, such as the Dysphagia Symptom Diary and Numeric Rating Scales for Dysphagia and Pain, respectively, have been used in other drug development programs, as historically licensing DSQ to all interested parties has not been possible.^14,51^ The use of different instruments in different clinical trials poses challenges for evidence synthesis and impedes study cross-comparison. Therefore, even though instruments such as EEsAI PRO do not use a 24-hour recall, they may continue to be used as secondary end points to allow for comparisons with existing data or when implementation of a daily electronic diary poses challenges for investigator-initiated studies. No specific instruments reached consensus for use in observational studies. This likely reflects the different logistical challenges and heterogeneity in observational trials, wherein daily or extensive assessments may not be feasible, and many of the instruments proposed remain proprietary.

The development of a generic daily recall instrument was identified as a priority because existing tools such as DSQ and episode-based instruments may be difficult or expensive to implement outside of industry-sponsored RCTs. Whether such instruments should use broad language to describe dysphagia is another relevant consideration and was a subject of much debate. Currently, most available instruments do not assess all possible symptoms relevant for adults with EoE and do not include the most common language used by patients to describe dysphagia (food being stuck, delayed passage of food, tightness, and trouble swallowing based on qualitative work).^19,52^ “Food being stuck” narrowly missed the consensus criteria during the ratification round because there were concerns raised that this more accurately reflected food bolus impaction rather than dysphagia, although no clear distinction between language used to describe short- and long-lasting episodes of dysphagia has been noted in qualitative work. Last, data on cross-comparisons of instruments are scarce, and it is not clear whether assessing symptoms more broadly by including all possible dysphagia language as well as all symptom domains relevant to patients might better explain the variation in severity of biologic findings compared to assessing dysphagia frequency alone.^53,54^

The PEESS v2.0 is the only currently available instrument for assessing symptoms in pediatric patients with EoE. This tool was studied and validated in pediatric patients ≥8 years of age, as well as by parent proxy in patients ≥2 years of age and older. Although there are data to convincingly demonstrate the alignment between patient-reported and proxy-reported symptom severity, there is not enough data to understand the performance of this instrument in the context of treatment response, especially given the following: (1) there is a 30-day recall period for this instrument; (2) age influences symptom presentation in children, often without true dysphagia; and (3) a broad range of symptoms needs to be assessed.^25,55,56^ Health-related QoL is frequently assessed in children with EoE using the PedsQL. Health-related QoL scores are associated with EoE symptom scores and improve after treatment.^57,58^ While assessment of general health–related QoL allows for comparisons across other diseases, there was debate about the utility of assessing general health–related QoL in pediatric patients rather than disease-specific QoL, leading to the exclusion of this measure from the COS.

Our study has several strengths. We used rigorous methods to develop this COS; each method had unique strengths. For example, anonymous online Delphi surveys allowed us to capture a large panel of international experts, whereas in-person live discussions highlighted more nuanced arguments for or against specific outcomes. However, we also acknowledge some important limitations. First, there are some outcomes included in the COS that appear to be inconsistent (for example, reporting both eos/hpf and eos/mm^2^; reporting PEC of <15 eos/hpf vs ≤6 eos/hpf). This typically reflects insufficient empirical evidence to guide decision making, and in these scenarios, we have recommended that both measures be reported. Nevertheless, we realize this recommendation does not remove an ambiguity with respect to reporting of trial results, especially with regard to measures of spread, which are not easily converted between units. Collecting these data will facilitate comparative analyses that can inform future iterations of the COS. Second, we restricted the COS to measures of treatment efficacy or effectiveness, rather than safety outcomes. Given the diverse drug targets under investigation, which have different safety profiles from conventional corticosteroids and dietary therapies, it was thought that proscribing adverse event reporting was outside the scope of this COS. Third, we engaged patients for deciding the outcome domains of importance. However, patients were recruited from a single country, and there was limited racial/ethnic diversity. Nevertheless, almost all patients included in this study identified similar outcome domains of importance, which made it unlikely that these would be dropped from later rounds of the Delphi process. Additionally, specific patient input on measurement tools was not sought because these decisions were primarily based on technical factors. For example, while we thought it was critical to assess patient perceptions of endoscopic evaluation as an outcome, the specific considerations regarding whether the EREFS should be scored on a 0–8 versus 0–9 scale were less relevant for patients. Fourth, some domains, such as patients’ perception of health or secondary impact on caregivers, were likely voted as subjects of future research by the experts because of limited data currently available in these areas. Fifth, we recognize that we included authors who have been pivotal in developing instruments that are advocated for in this COS. However, we thought it was important to capture the expertise of the global EoE community. Finally, we did not engage industry stakeholders because this was an academic exercise, and we did not engage regulators because they are generally precluded from these types of initiatives as a result of potential conflicts of interest.

In conclusion, we have developed an internationally guided COS for use in pharmacologic and dietary therapeutic trials in pediatric and adult patients with EoE. Groups assessing EoE therapies should be encouraged to adopt this COS to reduce the heterogeneity in outcome reporting and to improve comparability to future studies. We recognize that the end points used in EoE trials have evolved rapidly over the past 2 decades. Although this is the first iteration of a COS in EoE, we anticipate that ongoing work in the development and validation of new instruments for measuring disease activity will shape both future versions of this COS and the field moving forward.

## Supplementary Material

E2

E3

E1

## Figures and Tables

**TABLE I.** Demographic characteristics of the expert panel

Characteristic	Outcome domains (n = 66)	Delphi round 1 survey (n = 69)	Delphi round 2 survey (n = 62)	Ratification meeting (n = 27)

Specialty				
Gastroenterology	33 (50.0)	38 (55.1)	35 (56.5)	16 (59.3)
Allergy	16 (24.2)	14 (20.3)	12 (19.4)	2 (7.4)
Pathology	11 (16.7)	10 (14.5)	8 (12.9)	5 (18.5)
Other	6 (9.1)	7 (10.1)	7 (11.3)	4 (14.8)
Patient population				
Adult only (≥18 years)	31 (47.0)	32 (46.4)	31 (50.0)	13 (48.1)
Both adult and pediatric	17 (25.8)	19 (27.5)	18 (29.0)	7 (25.9)
Pediatric only (<18 years)	18 (27.3)	18 (26.1)	13 (21.0)	7 (25.9)
Practice setting				
Academic hospital/clinic	58 (87.9)	60 (87.0)	54 (87.1)	23 (85.2)
Nonacademic hospital/clinic	8 (12.1)	9 (13.0)	8 (12.9)	4 (14.8)
Geographic region				
United States	37 (56.1)	40 (58.0)	35 (56.5)	15 (55.6)
Europe	25 (37.9)	24 (34.8)	23 (37.1)	8 (29.6)
Other	4 (6.1)	5 (7.2)	4 (6.5)	4 (14.8)

Data are presented as no. (%).

**TABLE II.** Voting distribution on a 9-point Likert scale for the importance of different outcome domains for inclusion in a core outcome set for eosinophilic esophagitis

Outcome domain	Not important for inclusion (1–3)	Important but not critical for inclusion (4–6)	Critical for inclusion (7–9)

Histology	0	2 (3.0)	65 (97.0)
Endoscopy	1 (1.5)	3 (4.6)	61 (93.8)
Patient-reported symptoms	0	6 (9.1)	60 (90.9)
EoE-specific quality of life	1 (1.6)	15 (23.4)	48 (75.0)
Biomarkers	6 (9.2)	30 (46.2)	29 (44.6)
Esophageal distensibility	3 (4.9)	33 (54.1)	25 (41.0)
Genetic profiling	19 (29.7)	28 (43.8)	17 (26.6)
Immunologic dissection	14 (21.2)	37 (56.1)	15 (22.7)
Patient perception of health	1 (1.6)	34 (53.1)	29 (45.3)
Secondary impact on caregivers	10 (15.6)	39 (60.9)	15 (23.4)
Resource utilization	14 (23.7)	33 (55.9)	12 (20.3)

Data are presented as no. (%).

**TABLE III.** Core outcome set for eosinophilic esophagitis

Outcome domain	RCTs	Observational studies

Histopathology	Peak esophageal eosinophilia (and appropriate measures of spread, such as error terms or confidence intervals) should be measured and reported in all RCTs, expressed as: • No. of eosinophils per high-power field (400× magnification). • No. of cells adjusted per mm^2^ (400× magnification). Histologic remission should be measured in all RCTs. • In RCTs, histologic remission should be defined on the basis of a PEC of <15 esophageal eos/hpf in any location.^a^ Grade (severity) and stage (extent) of all components in EoEHSS should be measured and reported in all RCTs. • EoEHSS remission score should be measured and reported in all RCTs; for each item, proximal and distal esophagus: remission score of ≤3 for grade AND ≤3 for stage AND PEC of <15 eos/hpf.	Peak esophageal eosinophilia (and appropriate measures of spread, such as error terms or confidence intervals) should be measured and reported in all observational studies, expressed as: • No. of eosinophils per high-power field (400× magnification). • No. of cells adjusted per mm^2^ (400× magnification). Histologic remission should be measured in all observational studies. • In observational studies, histologic remission should be defined on the basis of a PEC of <15 esophageal eos/hpf in any location.^a^
Endoscopy	EREFS should be measured and reported in all RCTs. • EREFS should be scored from 0 to 8, scoring the most severe grade of esophageal EoE-associated features present in the proximal and distal esophagus (with furrows scored as absent or present).^b^	EREFS should be measured and reported in all observational studies. • EREFS should be scored from 0 to 8, scoring the most severe grade of esophageal EoE-associated features present in the proximal and distal esophagus (with furrows scored as absent or present).^b^
	Endoscopic remission based on EREFS should be measured and reported in all RCTs and observational studies. • In RCTs or observational studies, the endoscopic EREFS-based remission should be defined as an EREFS score of ≥2 (based on EREFS scoring from 0 to 8).^c^ • In RCTs or observational studies, endoscopic inflammatory EREFS-based remission should be defined as inflammation-associated components (exudate, edema, furrows) score of ≤2 (based on EREFS scoring from 0 to 8). • In RCTs or observational studies, the endoscopic fibrotic EREFS-based remission should be defined as categorical definition as absence of strictures, and moderate and severe rings.	
Patient-reported symptoms	In all RCTs, symptom severity in adults with EoE should be assessed using a generic instrument with a daily recall period.^d^ In all RCTs, symptom severity in adults with EoE should be assessed using the following instruments: • Dysphagia Symptom Questionnaire. • Eosinophilic Esophagitis Activity Index (7-day recall period). In all RCTs, the following language should be used to query dysphagia in adults with EoE: • Dysphagia defined as trouble swallowing. • Dysphagia defined as delayed or slow passage of food. In all RCTs, symptom severity in pediatric EoE patients should be measured using PEESS v2.0.	No patient-reported symptom instruments met consensus thresholds for use in all observational studies. In all observational studies, the following language should be used to query dysphagia in adults with EoE: • Dysphagia defined as trouble swallowing. • Dysphagia defined as delayed or slow passage of food.
QoL	In all RCTs, EoE-specific QoL in adults should be measured using EoE QoL (EoE-QoL-A) questionnaire. In all RCTs, pediatric EoE-specific QoL should be measured using PedsQL EoE module. • When using PedsQL EoE Module for children, for whom both parent-proxy report and child self-report are available, both should be reported in all RCTs.	No patient-reported QoL instruments met consensus thresholds for use in all observational studies.

*EoE-QoL-A*, Adult Eosinophilic Oesophagitis Quality of Life; *eos/hpf*, eosinophils per high-power field.

a Remission cutoff of <15 eos/hpf corresponding to <60 eosinophils/mm^2^.

b If the EREFS is scored on a 0–9 scale, it is recommended to report component scores to calculate *post hoc* an EREFS score on a 0–8 scale.

c Endoscopic remission recommended to be defined by EREFS ≤ 2 if scored on a 0–8 or 0–9 scale.

d Considered appropriate to use a generic instrument with a daily recall period in accordance with regulatory recommendations.

## Key messages

- Developing a core outcome set (COS), a minimum set of outcomes to be reported in all controlled and observational studies in children and adults with eosinophilic esophagitis (EoE), is important for improving clinical trial design and evidence synthesis.
- This international COS consensus exercise identified tools to be used to standardize disease activity assessment in EoE.

## Abbreviations used

AAAAI: American Academy of Allergy, Asthma & Immunology
CEGIR: Consortium of Eosinophilic Gastrointestinal Disease Researchers
COMET: Core Outcome Measures in Effectiveness Trials
COREOS: Core outcome set for therapeutic studies in eosinophilic esophagitis
COS: Core outcome set
DSQ: Dysphagia Symptoms Questionnaire
EEsAI: Eosinophilic Esophagitis Activity Index
EGID: Eosinophil Gastrointestinal Disorders
EoE: Eosinophilic esophagitis
EoEHSS: Eosinophilic Esophagitis Histology Scoring System
EREFS: Eosinophilic Esophagitis Endoscopic Reference Score
EUREOS: European Society of Eosinophilic Esophagitis
FDA: US Food and Drug Administration
hpf: High-power field
PEC: Peak eosinophil count
PedsQL: Pediatric Quality of Life Inventory
PEESS: Pediatric Eosinophilic Esophagitis Symptom Score
PRO: Patient-Reported Outcome
QoL: Quality of life
RCT: Randomized controlled trial
