# Supplementary material for: Development of a core outcome set for therapeutic studies in eosinophilic esophagitis (COREOS)
Source: J Allergy Clin Immunol. Author manuscript; Available in PMC 2023 Feb 1. (PMC8733049; doi:10.1016/j.jaci.2021.07.001)
Supplement: E2 [file NIHMS1740712-supplement-E2.pdf]

**Supplemental Table 2.** Results from Round 2 survey.

| Item | Statements | Votes (n) | Median | 1 – 3 n (%) | 4 – 6 n %) | 7 – 9 n (%) |
|---|---|---|---|---|---|---|
| **1** | In RCTs, PEC should be assessed as eos/hpf | 58 | 9 | 1 ( 1.7%) | 9 (15.5%) | 48 (82.8%) |
| **2** | In RCTs, PEC should be assessed as eos/mm2 hpf | 61 | 9 | 2 ( 3.3%) | 9 (14.8%) | 50 (82.0%) |
| **3** | In OBS, PEC should be assessed as eos/hpf | 58 | 8 | 1 ( 1.7%) | 6 (10.3%) | 51 (87.9%) |
| **4** | In OBS, PEC should be assessed as eos/mm2 hpf | 61 | 8 | 4 ( 6.6%) | 8 (13.1%) | 49 (80.3%) |
| **5** | Histologic remission should be measured in all RCTs | 62 | 9 | 0 (0%) | 1 ( 1.6%) | 61 (98.4%) |
| **6** | In RCTs, histologic remission should be defined as PEC ≤6/hpf in any location | 58 | 7.5 | 2 ( 3.4%) | 14 (24.1%) | 42 (72.4%) |
| **7** | In RCTs, histologic remission should be defined as PEC <15/hpf in any location | 57 | 8 | 6 (10.5%) | 11 (19.3%) | 40 (70.2%) |
| **8** | In RCTs, histologic remission should be defined as PEC ≤25/mm2 hpf in any location | 55 | 7 | 13 (23.6%) | 13 (23.6%) | 29 (52.7%) |
| **9** | In RCTs, histologic remission should be defined as PEC <60/mm2 hpf in any location | 55 | 6 | 18 (32.7%) | 10 (18.2%) | 27 (49.1%) |
| **10** | Histologic remission should be measured in all OBS | 61 | 9 | 0 (0%) | 6 ( 9.8%) | 55 (90.2%) |
| **11** | In OBS, histologic remission should be defined as PEC ≤6/hpf in any location | 58 | 7 | 4 ( 6.9%) | 18 (31.0%) | 36 (62.1%) |
| **12** | In OBS, histologic remission should be defined as PEC <15/hpf in any location | 57 | 8 | 6 (10.5%) | 12 (21.1%) | 39 (68.4%) |
| **13** | In OBS, histologic remission should be defined as PEC ≤25/mm2 hpf in any location | 57 | 7 | 13 (22.8%) | 15 (26.3%) | 29 (50.9%) |
| **14** | In OBS, histologic remission should be defined as PEC <60/mm2 hpf in any location | 55 | 6 | 18 (32.7%) | 14 (25.5%) | 23 (41.8%) |
| **15** | The grade (severity) and stage (extent) of all components in the EoEHSS should be measured in all RCTs | 60 | 8 | 1 ( 1.7%) | 11 (18.3%) | 48 (80.0%) |
| **16** | The EoEHSS remission score should be measured in all RCTs (For EACH proximal and distal esophagus: ≤3 for grade AND ≤3 for stage AND PEC of <15 eos/hpf) | 59 | 8 | 2 ( 3.4%) | 11 (18.6%) | 46 (78.0%) |
| **17** | Only eosinophil inflammation grade should be measured in all OBS | 55 | 7 | 4 ( 7.3%) | 20 (36.4%) | 31 (56.4%) |
| **18** | Only eosinophil inflammation stage should be measured in all OBS | 57 | 6 | 5 ( 8.8%) | 25 (43.9%) | 27 (47.4%) |
| **19** | The EREFS should be measured and reported in all RCTs | 58 | 9 | 0 (0%) | 1 ( 1.7%) | 57 (98.3%) |
| **20** | The EREFS should be measured and reported in all OBS | 58 | 8 | 1 ( 1.7%) | 12 (20.7%) | 45 (77.6%) |
| **21** | In RCTs, EREFS should be scored from 0 to 8, scoring the most severe grade of esophageal EoE-associated features present in proximal and distal esophagus (furrows scored as absent and present) | 53 | 8 | 0 (0%) | 4 ( 7.5%) | 49 (92.5%) |
| **22** | In OBS, EREFS should be scored from 0 to 8, scoring the most severe grade of esophageal EoE-associated features present in proximal and distal esophagus (furrows scored as absent and present) | 53 | 8 | 2 ( 3.8%) | 15 (28.3%) | 36 (67.9%) |
| **23** | Endoscopic remission based on EREFS should be measured and reported in all RCTs | 55 | 9 | 0 (0%) | 3 ( 5.5%) | 52 (94.5%) |
| **24** | In RCTs, the endoscopic EREFS-based remission should be defined as score ≤2 (total score 0 to 8, scoring the most severe grade of esophageal EoE-associated features present in proximal and distal esophagus) | 52 | 8 | 0 (0%) | 8 (15.4%) | 44 (84.6%) |

| Item | Statements | Votes (n) | Median | 1 – 3 n (%) | 4 – 6 n %) | 7 – 9 n (%) |
|---|---|---|---|---|---|---|
| **25** | In RCTs, the endoscopic inflammatory EREFS-based remission should be defined as the inflammation-associated components EEF score ≤2 (total score 0 to 8, scoring the most severe grade of esophageal EoE-associated features present in proximal and distal esophagus) | 49 | 8 | 1 ( 2.0%) | 10 (20.4%) | 38 (77.6%) |
| **26** | In RCTs, the endoscopic fibrotic EREFS-based remission should be defined as categorical definition as absence of strictures, moderate and severe rings | 51 | 8 | 0 (0%) | 10 (19.6%) | 41 (80.4%) |
| **27** | Endoscopic remission based on EREFS should be measured and reported in all OBS | 56 | 8 | 2 ( 3.6%) | 17 (30.4%) | 37 (66.1%) |
| **28** | In OBS, the endoscopic EREFS-based remission should be defined as score ≤2 (total score 0 to 8, scoring the most severe grade of esophageal EoE-associated features present in proximal and distal esophagus) | 49 | 7 | 2 ( 4.1%) | 13 (26.5%) | 34 (69.4%) |
| **29** | In OBS, the endoscopic inflammatory EREFS-based remission should be defined as the inflammation-associated components EEF score ≤2 (total score 0 to 8, scoring the most severe grade of esophageal EoE-associated features present in proximal and distal esophagus) | 48 | 7 | 2 ( 4.2%) | 18 (37.5%) | 28 (58.3%) |
| **30** | In OBS, the endoscopic fibrotic EREFS-based remission should be defined as categorical definition as absence of strictures, moderate and severe rings | 50 | 7 | 1 ( 2.0%) | 15 (30.0%) | 34 (68.0%) |
| **31** | In all RCTs, symptom severity in adults with EoE should be assessed using the Dysphagia Symptom Questionnaire | 45 | 7 | 0 (0%) | 14 (31.1%) | 31 (68.9%) |
| **32** | In all RCTs, symptom severity in adults with EoE should be assessed using the Dysphagia Symptom Diary | 45 | 7 | 3 ( 6.7%) | 18 (40.0%) | 24 (53.3%) |
| **33** | In all RCTs, symptom severity in adults with EoE should be assessed using the Eosionophilic Esophagitis Activity Index (7-day recall period) | 47 | 8 | 1 ( 2.1%) | 6 (12.8%) | 40 (85.1%) |
| **34** | In all RCTs, symptom severity in adults with EoE should be assessed using the Numeric Rating Scale for dysphagia | 46 | 7 | 3 ( 6.5%) | 19 (41.3%) | 24 (52.2%) |
| **35** | In all RCTs, symptom severity in adults with EoE should be assessed using the Numeric Rating Scale for pain | 45 | 6 | 7 (15.6%) | 16 (35.6%) | 22 (48.9%) |
| **36** | In all RCTs, symptom severity in adults with EoE should be assessed using the Visual Analogue Scale for dysphagia severity | 45 | 5 | 6 (13.3%) | 25 (55.6%) | 14 (31.1%) |
| **37** | In all RCTs, symptom severity in adults with EoE should be assessed using the Episode-based patient-reported outcome measure of dysphagia experience used in the FLUTE study | 45 | 6 | 5 (11.1%) | 25 (55.6%) | 15 (33.3%) |
| **38** | In all RCTs, the trouble swallowing should be used as language to query dysphagia in adults with EoE | 50 | 8 | 1 ( 2.0%) | 2 ( 4.0%) | 47 (94.0%) |
| **39** | In all RCTs, delayed or slow passage of food should be used as language to query dysphagia in adults with EoE | 52 | 9 | 1 ( 1.9%) | 4 ( 7.7%) | 47 (90.4%) |
| **40** | In all RCTs, food being stuck should be used as language to query dysphagia in adults with EoE | 51 | 8 | 5 ( 9.8%) | 6 (11.8%) | 40 (78.4%) |
| **41** | In all OBS, symptom severity in adults with EoE should be assessed using the Dysphagia Symptom Questionnaire | 45 | 7 | 2 ( 4.4%) | 19 (42.2%) | 24 (53.3%) |
| **42** | In all OBS, symptom severity in adults with EoE should be assessed using the Dysphagia Symptom Diary | 46 | 6 | 6 (13.0%) | 22 (47.8%) | 18 (39.1%) |
| **43** | In all OBS, symptom severity in adults with EoE should be assessed using the Eosionophilic Esophagitis Activity Index (7-day recall period) | 46 | 7 | 1 ( 2.2%) | 18 (39.1%) | 27 (58.7%) |

| Item | Statements | Votes (n) | Median | 1 – 3 n (%) | 4 – 6 n %) | 7 – 9 n (%) |
|---|---|---|---|---|---|---|
| **44** | In all OBS, symptom severity in adults with EoE should be assessed using the Numeric Rating Scale for dysphagia | 44 | 6 | 6 (13.6%) | 19 (43.2%) | 19 (43.2%) |
| **45** | In all OBS, symptom severity in adults with EoE should be assessed using the Numeric Rating Scale for pain | 44 | 5.5 | 7 (15.9%) | 23 (52.3%) | 14 (31.8%) |
| **46** | In all OBS, symptom severity in adults with EoE should be assessed using the Visual Analogue Scale for dysphagia severity | 44 | 5.5 | 7 (15.9%) | 22 (50.0%) | 15 (34.1%) |
| **47** | In all OBS, the trouble swallowing should be used as language to query dysphagia in adults with EoE | 51 | 8 | 2 ( 3.9%) | 7 (13.7%) | 42 (82.4%) |
| **48** | In all OBS, delayed or slow passage of food should be used as language to query dysphagia in adults with EoE | 52 | 9 | 1 ( 1.9%) | 7 (13.5%) | 44 (84.6%) |
| **49** | In all OBS, food being stuck should be used as language to query dysphagia in adults with EoE | 51 | 8 | 4 ( 7.8%) | 12 (23.5%) | 35 (68.6%) |
| **50** | In all RCTs, EoE-specific quality of life in adults should be measured using EoE-QOL-A questionnaire | 49 | 8 | 2 ( 4.1%) | 7 (14.3%) | 40 (81.6%) |
| **51** | In all OBS, EoE-specific quality of life in adults should be measured using EoE-QOL-A questionnaire | 49 | 7 | 2 ( 4.1%) | 16 (32.7%) | 31 (63.3%) |
| **52** | In all RCTs, symptom severity in pediatric EoE patients should be measured using PEESS v2.0 | 46 | 8 | 0 (0%) | 8 (17.4%) | 38 (82.6%) |
| **53** | In all OBS, symptom severity in pediatric EoE patients should be measured using PEESS v2.0 | 46 | 7 | 0 (0%) | 17 (37.0%) | 29 (63.0%) |
| **54** | In all RCTs, pediatric health-related quality of life should be measured using PedsQL | 44 | 8 | 2 ( 4.5%) | 7 (15.9%) | 35 (79.5%) |
| **55** | When using generic PedsQL for children of ages, for whom both parent-proxy (PR) report and child self-report (CR) are available, both should be reported in all RCTs | 43 | 8 | 2 ( 4.7%) | 9 (20.9%) | 32 (74.4%) |
| **56** | In all RCTs, pediatric EoE-specific quality of life should be measured using PedsQL EoE Module | 42 | 8 | 1 ( 2.4%) | 6 (14.3%) | 35 (83.3%) |
| **57** | When using PedsQL EoE Module for children of ages, for whom both parent-proxy (PR) report and child self-report (CR) are available, both should be reported in all RCTs | 43 | 8 | 1 ( 2.3%) | 11 (25.6%) | 31 (72.1%) |
| **58** | In all OBS, pediatric EoE-specific quality of life should be measured using PedsQL EoE Module | 43 | 7 | 2 ( 4.7%) | 15 (34.9%) | 26 (60.5%) |
| **59** | When using PedsQL EoE Module for children of ages, for whom both parent-proxy (PR) report and child self-report (CR) are available, both should be reported in all OBS | 44 | 7 | 3 ( 6.8%) | 17 (38.6%) | 24 (54.5%) |
