# Supplementary material for: Development of a core outcome set for therapeutic studies in eosinophilic esophagitis (COREOS)
Source: J Allergy Clin Immunol. Author manuscript; Available in PMC 2023 Feb 1. (PMC8733049; doi:10.1016/j.jaci.2021.07.001)
Supplement: E3 [file NIHMS1740712-supplement-E3.pdf]

Supplemental Table 3. Results from ratification round survey.

Item	Statement	Include (n)	Include (%)	Unsure (n)	Unsure (%)	Do not include (n)	Do not include (%)	Sum
**1**	Peak esophageal eosinophilia (AND appropriate measures of spread, such as error terms, confidence intervals) should be measured and reported in all RCTs and expressed as: number of cells per high-power field (400 × magnification)	22	100%	0	0%	0	0%	22
**2**	In RCTs, PEC should be assessed as number of cells adjusted per mm2 high-power field (400 × magnification)	21	96%	0	0%	1	5%	22
**3**	Peak esophageal eosinophilia (AND appropriate measures of spread, such as error terms, confidence intervals) should be measured and reported in all observational studies and expressed as: Number of cells per high-power field (400 × magnification)	20	91%	2	9%	0	0%	22
**4**	In observational studies, PEC should be assessed as number of cells adjusted per mm2 high-power field (400 × magnification)	19	86%	1	5%	2	9%	22
**5**	Histologic remission should be measured in all RCTs	24	100%	0	0%	0	0%	24
**6**	In RCTs, histologic remission should be defined based on the following peak esophageal eosinophilia cut-off: a peak of ≤ 6 esophageal eosinophils per high-power field in any location	13	54%	7	29%	4	17%	24
**7**	In RCTs, histologic remission should be defined based on the following peak esophageal eosinophilia cut-off: a peak of < 15 esophageal eosinophils per high-power field in any location	18	75%	4	17%	2	8%	24
**8**	Histologic remission should be measured in all observational studies	24	100%	0	0%	0	0%	24
**9**	In all observational studies, histologic remission should be defined based on the following peak esophageal eosinophilia cut-off: a peak of ≤ 6 esophageal eosinophils per high-power field in any location	8	33%	8	33%	8	33%	24
**10**	In all observational studies, histologic remission should be defined based on the following peak esophageal eosinophilia cut-off: a peak of < 15 esophageal eosinophils per high-power field in any location	24	100%	0	0%	0	0%	24
**11**	The grade (severity) and stage (extent) of all components in the EoEHSS (EoE Histologic Scoring System) should be measured in all RCTs	23	96%	1	4%	0	0%	24
**12**	The EoEHSS (EoE Histologic Scoring System) remission score should be measured in all RCTs: for EACH proximal and distal esophagus: remission score of ≤3 for grade AND ≤ 3 for stage AND peak eosinophil count of < 15 eos/hpf	20	83%	3	13%	1	4%	24
**13**	The Endoscopic Reference Score (EREFS) should be measured and reported in all RCTs	22	100%	0	0%	0	0%	22
**14**	The Endoscopic Reference Score (EREFS) should be measured and reported in all observational studies	18	82%	4	18%	0	0%	22

Item	Statement	Include (n)	Include (%)	Unsure (n)	Unsure (%)	Do not include (n)	Do not include (%)	Sum
**15**	If using the Endoscopic Reference Score (EREFS) in RCTs, the EREFS should be scored from 0 to 8, scoring the most severe grade of esophageal EoE-associated features present in proximal and distal esophagus (furrows scored as absent and present)	20	91%	2	9%	0	0%	22
**16**	If using the EREFS in observational studies, the EREFS should be scored from 0 to 8, scoring the most severe grade of esophageal EoE-associated features present in proximal and distal esophagus (furrows scored as absent and present)	19	86%	3	14%	0	0%	22
**17**	Endoscopic remission based on EREFS should be measured and reported in all RCTs	19	95%	1	5%	0	0%	20
**18**	In RCTs, the endoscopic EREFS-based remission should be defined as the EREFS score ≤2 (total score 0 to 8, scoring the most severe grade of esophageal EoE-associated features present in proximal and distal esophagus)	19	95%	1	5%	0	0%	20
**19**	In RCTs, the endoscopic inflammatory EREFS-based remission should be defined as the inflammation-associated components EEF score ≤2 (total score 0 to 8, scoring the most severe grade of esophageal EoE-associated features present in proximal and distal esophagus)	17	85%	2	10%	1	5%	20
**20**	In RCTs, the endoscopic fibrotic EREFS-based remission should be defined as categorical definition as absence of strictures, moderate and severe rings	17	85%	2	10%	1	5%	20
**21**	Endoscopic remission based on EREFS should be measured and reported in all observational studies	19	95%	1	5%	0	0%	20
**22**	In observational studies, the endoscopic EREFS-based remission should be defined as score ≤2 (total score 0 to 8, scoring the most severe grade of esophageal EoE-associated features present in proximal and distal esophagus)	18	90%	2	10%	0	0%	20
**23**	In observational studies, the endoscopic inflammatory EREFS-based remission should be defined as the inflammation-associated components score ≤2 (total 0 to 8, scoring most severe grade of esophageal EoE-associated features present in proximal and distal)	16	80%	3	15%	1	5%	20
**24**	In observational studies, the endoscopic fibrotic EREFS-based remission should be defined as categorical definition as absence of strictures, moderate and severe rings	16	80%	3	15%	1	5%	20
**25**	In all RCTs, symptom severity in adults with EoE should be assessed using the following instruments: the Dysphagia Symptom Questionnaire	14	74%	5	26%	0	0%	19
**26**	In all RCTs, symptom severity in adults with EoE should be assessed using the following instruments: the Eosinophilic Esophagitis Activity Index (7-day recall period)	14	74%	5	26%	0	0%	19
**27**	In all RCTs, the following language should be used to query dysphagia in adults with EoE: Dysphagia defined as trouble swallowing	16	84%	2	11%	1	5%	19

Item	Statement	Include (n)	Include (%)	Unsure (n)	Unsure (%)	Do not include (n)	Do not include (%)	Sum
**28**	In all RCTs, the following language should be used to query dysphagia in adults with EoE: Dysphagia defined as delayed or slow passage of food	14	74%	5	26%	0	0%	19
**29**	In all RCTs, the following language should be used to query dysphagia in adults with EoE: Dysphagia defined as food being stuck	13	68%	4	21%	2	11%	19
**30**	In all RCTs, symptom severity in adults with EoE should be assessed using a generic instrument with a daily recall period	14	70%	4	20%	2	10%	20
**31**	In all RCTs, symptom severity in adults with EoE should be assessed using the EEsAI (24 hour recall period)	11	58%	8	42%	0	0%	19
**32**	In all observational studies, symptom severity in adults with EoE should be assessed using the following instruments: the Eosionophilic Esophagitis Activity Index (7-day recall period)	9	45%	7	35%	4	20%	20
**33**	In all observational studies, the following language should be used to query dysphagia in adults with EoE: Dysphagia defined as trouble swallowing	17	85%	2	10%	1	5%	20
**34**	In all observational studies, the following language should be used to query dysphagia in adults with EoE: Dysphagia defined as delayed or slow passage of food	15	75%	4	20%	1	5%	20
**35**	In all observational studies, the following language should be used to query dysphagia in adults with EoE: Dysphagia defined as food being stuck	13	65%	1	5%	6	30%	20
**36**	In all RCTs, symptom severity in pediatric EoE patients should be measured using pediatric eosinophilic esophagitis symptom score (PEESS v2.0)	15	75%	5	25%	0	0%	20
**37**	In all observational studies, symptom severity in pediatric EoE patients should be measured using pediatric eosinophilic esophagitis symptom score (PEESS v2.0)	6	30%	11	55%	3	15%	20
**38**	In all RCTs, EoE-specific quality of life in adults should be measured using Eosinophilic Esophagitis Quality of Life (EoE-QOL-A) questionnaire	21	96%	1	5%	0	0%	22
**39**	In all observational studies, EoE-specific quality of life in adults should be measured using Eosinophilic Esophagitis Quality of Life (EoE-QOL-A) questionnaire	12	55%	4	18%	6	27%	22
**40**	In all RCTs, pediatric health-related quality of life should be measured using The Pediatric Quality of Life Inventory (PedsQL)	13	68%	6	32%	0	0%	19
**41**	When using generic PedsQL for children of ages, for whom both parent-proxy (PR) report and child self-report (CR) are available, both should be reported in all RCTs	11	58%	8	42%	0	0%	19
**42**	In all RCTs, pediatric EoE-specific quality of life should be measured using The Pediatric Quality of Life Inventory (PedsQL) Eosinophilic Esophagitis Module:	16	84%	3	16%	0	0%	19
**43**	When using PedsQL EoE Module for children of ages, for whom both parent-proxy (PR) report and child self-report (CR) are available, both should be reported in all RCTs	14	74%	5	26%	0	0%	19

Item	Statement	Include (n)	Include (%)	Unsure (n)	Unsure (%)	Do not include (n)	Do not include (%)	Sum
**44**	In all observational studies, pediatric EoE-specific quality of life should be measured using PedsQL EoE Module	7	37%	7	37%	5	26%	19
