# Supplementary material for: Development of a core outcome set for therapeutic studies in eosinophilic esophagitis (COREOS)
Source: J Allergy Clin Immunol. Author manuscript; Available in PMC 2023 Feb 1. (PMC8733049; doi:10.1016/j.jaci.2021.07.001)
Supplement: E1 [file NIHMS1740712-supplement-E1.pdf]

Supplemental Table 1. Results from Round 1 survey.

Item	Statements	Votes (n)	Median	1 – 3 n (%)	4 – 6 n (%)	7 – 9 n (%)
1	In RCTs, PEC should be assessed as eos/hpf	64	9	3 (4.7%)	10 (15.6%)	51 (79.7%)
2	In RCTs, PEC should be assessed as eos/mm2 hpf	66	9	4 (6.1%)	5 (7.6%)	57 (86.4%)
3	In OBS, PEC should be assessed as eos/hpf	66	8	2 (3.0%)	14 (21.2%)	50 (75.8%)
4	In OBS, PEC should be assessed as eos/mm2 hpf	67	8	7 (10.4%)	10 (14.9%)	50 (74.6%)
5	Histologic remission should be measured in all RCTs	69	9	0	1 (1.4%)	68 (98.6%)
6	In RCTs, histologic remission should be defined as PEC 0/hpf in any location	58	5	12 (20.7%)	30 (51.7%)	16 (27.6%)
7	In RCTs, histologic remission should be defined as PEC ≤6/hpf in any location	62	7	6 (9.7%)	14 (22.6%)	42 (67.7%)
8	In RCTs, histologic remission should be defined as PEC <15/hpf in any location	63	8	10 (15.9%)	11 (17.5%)	42 (66.7%)
9	In RCTs, histologic remission should be defined as PEC 0/mm2 hpf in any location	59	5	13 (22.0%)	28 (47.5%)	18 (30.5%)
10	In RCTs, histologic remission should be defined as PEC ≤25/mm2 hpf in any location	62	7	8 (12.9%)	22 (35.5%)	32 (51.6%)
11	In RCTs, histologic remission should be defined as PEC <60/mm2 hpf in any location	59	6	16 (27.1%)	18 (30.5%)	25 (42.4%)
12	Histologic remission should be measured in all OBS	66	9	1 (1.5%)	5 (7.6%)	60 (90.9%)
13	In OBS, histologic remission should be defined as PEC 0/hpf in any location	59	5	8 (13.6%)	34 (57.6%)	17 (28.8%)
14	In OBS, histologic remission should be defined as PEC ≤6/hpf in any location	63	7	4 (6.3%)	20 (31.7%)	39 (61.9%)
15	In OBS, histologic remission should be defined as PEC <15/hpf in any location	62	8	7 (11.3%)	12 (19.4%)	43 (69.4%)
16	In OBS, histologic remission should be defined as PEC 0/mm2 hpf in any location	59	5	14 (23.7%)	31 (52.5%)	14 (23.7%)
17	In OBS, histologic remission should be defined as PEC ≤25/mm2 hpf in any location	63	6	8 (12.7%)	26 (41.3%)	29 (46.0%)
18	In OBS, histologic remission should be defined as PEC <60/mm2 hpf in any location	61	6	14 (23.0%)	21 (34.4%)	26 (42.6%)
19	The grade (severity) and stage (extent) of all components in the EoEHSS should be measured in all RCTs	67	8	2 (3.0%)	9 (13.4%)	56 (83.6%)
20	The EoEHSS remission score should be measured in all RCTs (For EACH proximal and distal esophagus: ≤3 for grade AND ≤3 for stage AND PEC of <15 eos/hpf)	62	8	2 (3.2%)	13 (21.0%)	47 (75.8%)
21	Only the stage (extent) of all components in the EoEHSS should be measured in all RCTs	54	5	13 (24.1%)	23 (42.6%)	18 (33.3%)
22	Only the grade (severity) of all components in the EoEHSS should be measured in all RCTs	56	5	11 (19.6%)	26 (46.4%)	19 (33.9%)

Item	Statements	Votes (n)	Median	1 – 3 n (%)	4 – 6 n (%)	7 – 9 n (%)
23	Only eosinophil inflammation grade should be measured in all RCTs	60	9	1 (1.7%)	10 (16.7%)	49 (81.7%)
24	Only eosinophil inflammation stage should be measured in all RCTs	57	8	1 (1.8%)	10 (17.5%)	46 (80.7%)
25	Only basal zone hyperplasia grade should be measured in all RCTs	60	8	2 (3.3%)	18 (30.0%)	40 (66.7%)
26	Only basal zone hyperplasia stage should be measured in all RCTs	57	7	3 (5.3%)	16 (28.1%)	38 (66.7%)
27	Only eosinophil abscess grade should be measured in all RCTs	61	8	4 (6.6%)	14 (23.0%)	43 (70.5%)
28	Only eosinophil abscess stage should be measured in all RCTs	58	7	4 (6.9%)	16 (27.6%)	38 (65.5%)
29	Only eosinophil surface layering grade should be measured in all RCTs	60	7	4 (6.7%)	21 (35.0%)	35 (58.3%)
30	Only eosinophil surface layering stage should be measured in all RCTs	58	7	4 (6.9%)	23 (39.7%)	31 (53.4%)
31	Only dilated intercellular spaces grade should be measured in all RCTs	61	7	6 (9.8%)	15 (24.6%)	40 (65.6%)
32	Only dilated intercellular spaces stage should be measured in all RCTs	59	7	6 (10.2%)	16 (27.1%)	37 (62.7%)
33	Only surface epithelial alterations grade should be measured in all RCTs	59	6	9 (15.3%)	23 (39.0%)	27 (45.8%)
34	Only surface epithelial alterations stage should be measured in all RCTs	58	6	8 (13.8%)	25 (43.1%)	25 (43.1%)
35	Only dyskeratotic epithelial cells grade should be measured in all RCTs	58	6	12 (20.7%)	23 (39.7%)	23 (39.7%)
36	Only dyskeratotic epithelial cells stage should be measured in all RCTs	57	5	12 (21.1%)	23 (40.4%)	22 (38.6%)
37	Only lamina propria fibrosis grade should be measured in all RCTs	61	8	1 (1.6%)	15 (24.6%)	45 (73.8%)
38	Only lamina propria fibrosis stage should be measured in all RCTs	58	8	2 (3.4%)	14 (24.1%)	42 (72.4%)
39	The grade (severity) and stage (extent) of all components in the EoEHSS should be measured in all OBS	64	6	8 (12.5%)	28 (43.8%)	28 (43.8%)
40	The EoEHSS remission score should be measured in all OBS (For EACH proximal and distal esophagus: ≤3 for grade AND ≤3 for stage AND PEC of <15 eos/hpf)	61	6	12 (19.7%)	27 (44.3%)	22 (36.1%)
41	Only the stage (extent) of all components in the EoEHSS should be measured in all OBS	55	5	14 (25.5%)	28 (50.9%)	13 (23.6%)
42	Only the grade (severity) of all components in the EoEHSS should be measured in all OBS	57	5	15 (26.3%)	28 (49.1%)	14 (24.6%)
43	Only eosinophil inflammation grade should be measured in all OBS	58	7	5 (8.6%)	13 (22.4%)	40 (69.0%)
44	Only eosinophil inflammation stage should be measured in all OBS	57	7	5 (8.8%)	16 (28.1%)	36 (63.2%)
45	Only basal zone hyperplasia grade should be measured in all OBS	57	6	9 (15.8%)	23 (40.4%)	25 (43.9%)
46	Only basal zone hyperplasia stage should be measured in all OBS	57	6	9 (15.8%)	25 (43.9%)	23 (40.4%)
47	Only eosinophil abscess grade should be measured in all OBS	58	6	9 (15.5%)	24 (41.4%)	25 (43.1%)
48	Only eosinophil abscess stage should be measured in all OBS	57	5	9 (15.8%)	25 (43.9%)	23 (40.4%)

Item	Statements	Votes (n)	Median	1 – 3 n (%)	4 – 6 n (%)	7 – 9 n (%)
49	Only eosinophil surface layering grade should be measured in all OBS	57	5	9 (15.8%)	30 (52.6%)	18 (31.6%)
50	Only eosinophil surface layering stage should be measured in all OBS	56	5	9 (16.1%)	31 (55.4%)	16 (28.6%)
51	Only dilated intercellular spaces grade should be measured in all OBS	58	6	10 (17.2%)	24 (41.4%)	24 (41.4%)
52	Only dilated intercellular spaces stage should be measured in all OBS	57	5	11 (19.3%)	25 (43.9%)	21 (36.8%)
53	Only surface epithelial alterations grade should be measured in all OBS	57	5	11 (19.3%)	30 (52.6%)	16 (28.1%)
54	Only surface epithelial alterations stage should be measured in all OBS	56	5	11 (19.6%)	31 (55.4%)	14 (25.0%)
55	Only dyskeratotic epithelial cells grade should be measured in all OBS	55	5	16 (29.1%)	30 (54.5%)	9 (16.4%)
56	Only dyskeratotic epithelial cells stage should be measured in all OBS	54	5	16 (29.6%)	29 (53.7%)	9 (16.7%)
57	Only lamina propria fibrosis grade should be measured in all OBS	58	6.5	6 (10.3%)	23 (39.7%)	29 (50.0%)
58	Only lamina propria fibrosis stage should be measured in all OBS	56	6.5	7 (12.5%)	21 (37.5%)	28 (50.0%)
59	The EREFS should be measured and reported in all RCTs	61	9	0	1 (1.6%)	60 (98.4%)
60	The EREFS should be measured and reported in all OBS	61	8	0	13 (21.3%)	48 (78.7%)
61	In RCTs, EREFS should be scored from 0 to 8, scoring the most severe grade of esophageal EoE-associated features present in proximal and distal esophagus (furrows scored as absent and present)	49	8	2 (4.1%)	14 (28.6%)	33 (67.3%)
62	In RCTs, EREFS should be scored from 0 to 9, scoring the most severe grade of esophageal EoE-associated features present in proximal and distal esophagus (furrows scored as absent, mild, and severe)	49	8	8 (16.3%)	9 (18.4%)	32 (65.3%)
63	In RCTs, EREFS should be scored from 0 to 16, separately scoring esophageal EoE-associated features present in the proximal and distal esophagus (furrows scored as absent and present)	48	6	7 (14.6%)	20 (41.7%)	21 (43.8%)
64	In RCTs, EREFS should be scored from 0 to 18, separately scoring esophageal EoE-associated features present in the proximal and distal esophagus (furrows scored as absent, mild, and severe)	49	6	14 (28.6%)	13 (26.5%)	22 (44.9%)
65	In OBS, EREFS should be scored from 0 to 8, scoring the most severe grade of esophageal EoE-associated features present in proximal and distal esophagus (furrows scored as absent and present)	49	7	3 (6.1%)	17 (34.7%)	29 (59.2%)
66	In OBS, EREFS should be scored from 0 to 9, scoring the most severe grade of esophageal EoE-associated features present in proximal and distal esophagus (furrows scored as absent, mild, and severe)	49	6	10 (20.4%)	16 (32.7%)	23 (46.9%)

Item	Statements	Votes (n)	Median	1 – 3 n (%)	4 – 6 n (%)	7 – 9 n (%)
67	In OBS, EREFS should be scored from 0 to 16, separately scoring esophageal EoE-associated features present in the proximal and distal esophagus (furrows scored as absent and present)	48	5	12 (25.0%)	25 (52.1%)	11 (22.9%)
68	In OBS, EREFS should be scored from 0 to 18, separately scoring esophageal EoE-associated features present in the proximal and distal esophagus (furrows scored as absent, mild, and severe)	49	5	16 (32.7%)	19 (38.8%)	14 (28.6%)
69	Endoscopic remission based on EREFS should be measured and reported in all RCTs	59	8	1 (1.7%)	5 (8.5%)	53 (89.8%)
70	In RCTs, the endoscopic EREFS-based remission should be defined as score ≤2 (total score 0 to 9, scoring the most severe grade of esophageal EoE-associated features present in proximal and distal esophagus)	48	7	4 (8.3%)	17 (35.4%)	27 (56.3%)
71	In RCTs, the endoscopic EREFS-based remission should be defined as score ≤2 (total score 0 to 18, scoring the most severe grade of esophageal EoE-associated features present in proximal and distal esophagus)	47	6	6 (12.8%)	22 (46.8%)	19 (40.4%)
72	In RCTs, the endoscopic EREFS-based remission should be defined as categorical definition: absence of strictures, exudates, furrows, and moderate and severe rings	46	6.5	9 (19.6%)	14 (30.4%)	23 (50.0%)
73	In RCTs, the endoscopic EREFS-based remission should be defined as categorical definition: absence of strictures, exudates, moderate and severe rings	44	5	13 (29.5%)	16 (36.4%)	15 (34.1%)
74	In RCTs, the endoscopic inflammatory EREFS-based remission should be defined as the inflammation-associated components EEF score ≤2 (total score 0 to 8, scoring the most severe grade of esophageal EoE-associated features present in proximal and distal esophagus)	50	7	4 (8.0%)	18 (36.0%)	28 (56.0%)
75	In RCTs, the endoscopic inflammatory EREFS-based remission should be defined as the inflammation-associated components EEF score ≤2 (total score 0 to 16, scoring the most severe grade of esophageal EoE-associated features present in proximal and distal esophagus)	50	6	10 (20.0%)	17 (34.0%)	23 (46.0%)
76	In RCTs, the endoscopic inflammatory EREFS-based remission should be defined as categorical definition as absence of exudates	47	6	14 (29.8%)	15 (31.9%)	18 (38.3%)
77	In RCTs, the endoscopic fibrotic EREFS-based remission should be defined as categorical definition as absence of strictures, moderate and severe rings	54	8	4 (7.4%)	10 (18.5%)	40 (74.1%)
78	Endoscopic remission based on EREFS should be measured and reported in all OBS	56	7.5	1 (1.8%)	13 (23.2%)	42 (75.0%)
79	In OBS, the endoscopic EREFS-based remission should be defined as score ≤2 (total score 0 to 9, scoring the most severe grade of esophageal EoE-associated features present in proximal and distal esophagus)	49	7	6 (12.2%)	18 (36.7%)	25 (51.0%)

Item	Statements	Votes (n)	Median	1 – 3 n (%)	4 – 6 n (%)	7 – 9 n (%)
80	In OBS, the endoscopic EREFS-based remission should be defined as score ≤2 (total score 0 to 18, scoring the most severe grade of esophageal EoE-associated features present in proximal and distal esophagus)	48	5	11 (22.9%)	23 (47.9%)	14 (29.2%)
81	In OBS, the endoscopic EREFS-based remission should be defined as categorical definition: absence of strictures, exudates, furrows, and moderate and severe rings	48	6	9 (18.8%)	18 (37.5%)	21 (43.8%)
82	In OBS, the endoscopic EREFS-based remission should be defined as categorical definition: absence of strictures, exudates, moderate and severe rings	45	6	11 (24.4%)	18 (40.0%)	16 (35.6%)
83	In OBS, the endoscopic inflammatory EREFS-based remission should be defined as the inflammation-associated components EEF score ≤2 (total score 0 to 8, scoring the most severe grade of esophageal EoE-associated features present in proximal and distal esophagus)	50	6	8 (16.0%)	18 (36.0%)	24 (48.0%)
84	In OBS, the endoscopic inflammatory EREFS-based remission should be defined as the inflammation-associated components EEF score ≤2 (total score 0 to 16, scoring the most severe grade of esophageal EoE-associated features present in proximal and distal esophagus)	50	5	14 (28.0%)	22 (44.0%)	14 (28.0%)
85	In OBS, the endoscopic inflammatory EREFS-based remission should be defined as categorical definition as absence of exudates	49	6	9 (18.4%)	21 (42.9%)	19 (38.8%)
86	In OBS, the endoscopic fibrotic EREFS-based remission should be defined as categorical definition as absence of strictures, moderate and severe rings	53	8	4 (7.5%)	15 (28.3%)	34 (64.2%)
87	In all RCTs, symptom severity in adults with EoE should be assessed using the Dysphagia Symptom Questionnaire	49	7	1 (2.0%)	17 (34.7%)	31 (63.3%)
88	In all RCTs, symptom severity in adults with EoE should be assessed using the Dysphagia Symptom Diary	47	7	4 (8.5%)	13 (27.7%)	30 (63.8%)
89	In all RCTs, symptom severity in adults with EoE should be assessed using the Eosionophilic Esophagitis Activity Index (7-day recall period)	49	7	3 (6.1%)	11 (22.4%)	35 (71.4%)
90	In all RCTs, symptom severity in adults with EoE should be assessed using the Numeric Rating Scale for dysphagia	47	7	5 (10.6%)	15 (31.9%)	27 (57.4%)
91	In all RCTs, symptom severity in adults with EoE should be assessed using the Numeric Rating Scale for pain	47	6	8 (17.0%)	19 (40.4%)	20 (42.6%)
92	In all RCTs, symptom severity in adults with EoE should be assessed using the Visual Analogue Scale for dysphagia severity	48	6	7 (14.6%)	22 (45.8%)	19 (39.6%)
93	In all RCTs, symptom severity in adults with EoE should be assessed using the Episode-based patient-reported outcome measure of dysphagia experience used in the FLUTE study	46	5	6 (13.0%)	27 (58.7%)	13 (28.3%)
94	In all RCTs, the trouble swallowing should be used as language to query dysphagia in adults with EoE	52	8	2 (3.8%)	6 (11.5%)	44 (84.6%)

Item	Statements	Votes (n)	Median	1 – 3 n (%)	4 – 6 n (%)	7 – 9 n (%)
95	In all RCTs, delayed or slow passage of food should be used as language to query dysphagia in adults with EoE	52	8	0	6 (11.5%)	46 (88.5%)
96	In all RCTs, food being stuck should be used as language to query dysphagia in adults with EoE	53	8	2 (3.8%)	12 (22.6%)	39 (73.6%)
97	In all RCTs, tightness should be used as language to query dysphagia in adults with EoE	51	5	14 (27.5%)	20 (39.2%)	17 (33.3%)
98	In all RCTs, feeling the food going down should be used as language to query dysphagia in adults with EoE	52	7	9 (17.3%)	14 (26.9%)	29 (55.8%)
99	In all OBS, symptom severity in adults with EoE should be assessed using the Dysphagia Symptom Questionnaire	49	7	5 (10.2%)	19 (38.8%)	25 (51.0%)
100	In all OBS, symptom severity in adults with EoE should be assessed using the Dysphagia Symptom Diary	47	6	8 (17.0%)	17 (36.2%)	22 (46.8%)
101	In all OBS, symptom severity in adults with EoE should be assessed using the Eosionophilic Esophagitis Activity Index (7-day recall period)	49	6	6 (12.2%)	19 (38.8%)	24 (49.0%)
102	In all OBS, symptom severity in adults with EoE should be assessed using the Numeric Rating Scale for dysphagia	46	6	7 (15.2%)	17 (37.0%)	22 (47.8%)
103	In all OBS, symptom severity in adults with EoE should be assessed using the Numeric Rating Scale for pain	46	5.5	8 (17.4%)	19 (41.3%)	19 (41.3%)
104	In all OBS, symptom severity in adults with EoE should be assessed using the Visual Analogue Scale for dysphagia severity	48	6	8 (16.7%)	23 (47.9%)	17 (35.4%)
105	In all OBS, symptom severity in adults with EoE should be assessed using the Episode-based patient-reported outcome measure of dysphagia experience used in the FLUTE study	46	5	11 (23.9%)	27 (58.7%)	8 (17.4%)
106	In all OBS, the trouble swallowing should be used as language to query dysphagia in adults with EoE	52	8	2 (3.8%)	7 (13.5%)	43 (82.7%)
107	In all OBS, delayed or slow passage of food should be used as language to query dysphagia in adults with EoE	52	8	0	9 (17.3%)	43 (82.7%)
108	In all OBS, food being stuck should be used as language to query dysphagia in adults with EoE	52	7	2 (3.8%)	14 (26.9%)	36 (69.2%)
109	In all OBS, tightness should be used as language to query dysphagia in adults with EoE	51	6	12 (23.5%)	24 (47.1%)	15 (29.4%)
110	In all OBS, feeling the food going down should be used as language to query dysphagia in adults with EoE	52	6	9 (17.3%)	19 (36.5%)	24 (46.2%)
111	In all RCTs, EoE-specific quality of life in adults should be measured using EoE-QOL-A questionnaire	55	8	1 (1.8%)	12 (21.8%)	42 (76.4%)

Item	Statements	Votes (n)	Median	1 – 3 n (%)	4 – 6 n (%)	7 – 9 n (%)
112	In all OBS, EoE-specific quality of life in adults should be measured using EoE-QOL-A questionnaire	54	7	6 (11.1%)	18 (33.3%)	30 (55.6%)
113	In all RCTs, symptom severity in pediatric EoE patients should be measured using PEESS v2.0	45	9	0	6 (13.3%)	39 (86.7%)
114	In all OBS, symptom severity in pediatric EoE patients should be measured using PEESS v2.0	45	7	1 (2.2%)	16 (35.6%)	28 (62.2%)
115	In all RCTs, pediatric health-related quality of life should be measured using PedsQL	42	7.5	1 (2.4%)	12 (28.6%)	29 (69.0%)
116	When using generic PedsQL for children of ages, for whom both parent-proxy (PR) report and child self-report (CR) are available, both should be reported in all RCTs	43	8	1 (2.3%)	11 (25.6%)	31 (72.1%)
117	In all RCTs, pediatric EoE-specific quality of life should be measured using PedsQL EoE Module	42	8	1 (2.4%)	12 (28.6%)	29 (69.0%)
118	When using PedsQL EoE Module for children of ages, for whom both parent-proxy (PR) report and child self-report (CR) are available, both should be reported in all RCTs	42	8	1 (2.4%)	9 (21.4%)	32 (76.2%)
119	In all OBS, pediatric health-related quality of life should be measured using PedsQL	42	6	5 (11.9%)	18 (42.9%)	19 (45.2%)
120	When using generic PedsQL for children of ages, for whom both parent-proxy (PR) report and child self-report (CR) are available, both should be reported in all OBS	44	7	4 (9.1%)	16 (36.4%)	24 (54.5%)
121	In all OBS, pediatric EoE-specific quality of life should be measured using PedsQL EoE Module	43	7	4 (9.3%)	14 (32.6%)	25 (58.1%)
122	When using PedsQL EoE Module for children of ages, for whom both parent-proxy (PR) report and child self-report (CR) are available, both should be reported in all OBS	44	7	4 (9.1%)	14 (31.8%)	26 (59.1%)
